# A Multiscale Electromagnetic Method for Damage Detection and Quantitative Characterization of Radar-Absorbing Coatings on Aircraft Skins

**DOI:** 10.3390/s26196357

**Published:** 2026-10-08

**Authors:** Yang Fang, Wenbin Ren, Yijun Liu, Wei Cui, Yongli Ma, Bin Liu, Yong Li, Zhenmao Chen

**Affiliations:** 1 State Key Laboratory for Strength and Vibration of Mechanical Structures, Shaanxi Engineering Research Center of NDT and Structural Integrity Evaluation, School of Aerospace Engineering, Xi’an Jiaotong University, Xi’an 710049, China; renwenbin@stu.xjtu.edu.cn (W.R.); yijun0227@stu.xjtu.edu.cn (Y.L.); yong.li@mail.xjtu.edu.cn (Y.L.); 2 National Key Laboratory of Electromagnetic Information Control and Effects, Shenyang 110037, China; cvqing@sina.com (W.C.); mayongli1305@163.com (Y.M.); 3 Shaanxi Special Equipment Inspection and Research Institute, Xi’an 710049, China; 19991202881@163.com

**Keywords:** radar-absorbing coating damage, multiscale electromagnetic detection, angular-domain scattering screening, local near-field imaging, quantitative defect characterization

## Abstract

This study proposes a multiscale electromagnetic method that combines far-field angular-domain analysis with local near-field imaging to detect damage in radar-absorbing coatings on aircraft surfaces. Using an intact state as a reference, continuous anomalous angular intervals and their principal anomalous azimuths are identified via robust statistical criteria, employing the electromagnetic scattering energy ratio (ESER), defined here as a normalized squared-field response ratio, and the azimuth difference factor. These angular characteristics, combined with target geometry and a predefined detection area, are used to determine the direction and azimuthal range of candidate near-field scanning windows. Subsequently, coherent back-projection is applied to the local near-field measurements. The centroid and equivalent electromagnetic dimensions of the differential response are estimated using complex differential imaging, multithreshold analysis, and squared-magnitude-weighted central second moments. Numerical analysis of a simplified aircraft-like model demonstrates angular-domain screening and local characterization of impact, coating loss and bulging. For the rectangular coating-loss simulation, the equivalent electromagnetic length and width deviate from the nominal side lengths by 8.6% and 2.3%, respectively. The corresponding experimental deviations for localized 1 cm × 1 cm coating loss on a coated conical specimen are 9.2% and 6.3%. These results support two-stage screening and local equivalent-scale extraction under the investigated geometry, material and reference-matching conditions.

## 1. Introduction

Radar-absorbing coatings on aircraft skins are functional layers designed to control electromagnetic scattering. During service, wear, impact, and environmental exposure can cause coating loss, debonding, or local changes in surface geometry. These changes modify the material distribution and electromagnetic boundary conditions, thereby affecting the microwave scattering response. This study investigates a multiscale inspection method that links large-area screening of candidate regions with local imaging and dimensional characterization.

Existing non-destructive testing methods for damage to aircraft microwave-absorbing coatings and composite structures mainly include optical, infrared, ultrasonic, and eddy current testing. Optical inspection can identify cracks, scratches, and morphological anomalies using surface images [1]; infrared thermal imaging can detect impact defects in composite materials based on differences in thermal response [2], and can further estimate the defect depth through thermal diffusion response [3]; active thermal imaging and image processing can also be used for the identification and evaluation of impact-damaged areas in curved composite layers [4]; ultrasonic scanning can be used for damage detection in composite reinforced structures [5], and air-coupled ultrasound can be used for non-contact characterization of impact defects in glass-fiber-reinforced polymer (GFRP) composites [6]; eddy current detection can identify damage by utilizing changes in electromagnetic induction response in conductive composite materials [7]. Related reviews compare the applicable objects, detection capabilities, and implementation conditions of different non-destructive testing techniques [8]. The above methods mainly provide information on the morphology, thermal properties, elastic waves, or electromagnetic induction of defects, and their detection results are not directly equivalent to the scattering changes in the target in the radar frequency band. Therefore, for damage detection of absorbing coatings, it is also necessary to link the spatial characterization of defects with microwave scattering response, and to take into account the sampling costs of large-scale screening and local fine detection. Microwave detection is sensitive to changes in material electromagnetic properties, structural thickness, and interface state. It is non-contact and has a certain penetration capability, making it suitable for surface and near-surface damage detection of composite materials and microwave absorbing structures. Existing research involves microwave detection of GFRP composite materials, near-field and far-field microwave imaging of reinforced composite plates, and defect detection and characterization of coated GFRP tubular structures [9,10,11]. Related reviews also summarize the application of microwave technology in the detection of composite materials and aerospace composite structures [12,13]. For direct microwave scattering imaging, Wu et al. combined microwave images with one-stage object detectors to identify damage in radar-absorbing materials [14]. In terms of microwave thermal imaging, Tu et al. used the local thermal response generated by microwave excitation to detect unbonded defects in the absorbing coating [15]. They subsequently developed a microwave thermal imaging system that combines long-pulse microwave thermography with microwave lock-in thermography for the inspection of radar-absorbing materials [16]. Zhu et al. further proposed a debonding detection method for absorbing materials based on gradient waveguide heating and microwave pulse thermal imaging [17]. Existing microwave inspection approaches include direct scattering imaging, image-based damage recognition and microwave thermography. Tu et al. [16] combine long-pulse and lock-in thermography, illustrating staged acquisition of defect information. More recently, Fang et al. [18] improve surface-damage detection in curved radar-absorbing materials through dual-channel scale-adaptive clutter suppression and confidence-derived gating; He et al. [19] use microwave thermal responses to detect debonding between radar-absorbing materials and carbon-fiber-reinforced polymer (CFRP) substrates; and Rahman et al. [20] analyze composite structures with embedded antennas using intact and damaged electromagnetic responses and near-field images. These studies emphasize image interpretation, interfacial thermal responses or local electromagnetic characterization. The present work further examines how angular anomalies in global scattering can be combined with geometric priors to plan local near-field measurements and connect that planning with spatial localization and equivalent-scale extraction.

From the perspective of direct scattering measurement, damage to absorbing coatings alters local material distribution, surface continuity, and electromagnetic boundary conditions, thereby causing changes in the amplitude and phase of the received scattered field. Far-field angular-domain scattering measurements can identify anomalous angular intervals by comparing the damaged state with an undamaged reference, but mainly provide candidate orientations or regions. Local near-field scanning can further obtain the spatial location and contour of the damage, but full-domain point-by-point scanning requires acquiring a large number of measurement points, resulting in low detection efficiency. Therefore, combining far-field angular-domain screening constrained by a reference with local near-field imaging can form a two-stage detection process from candidate region screening to local quantitative characterization.

To address the difference in sampling scales between wide-area inspection and local quantitative characterization of radar-absorbing coatings, this study proposes a two-stage electromagnetic inspection method in which far-field angular-domain anomalies guide local near-field scanning. The main contributions are as follows: (1) an intact/damaged response comparison and robust angular-domain anomaly extraction procedure is developed to identify continuous anomalous intervals and the principal anomalous azimuth, accounting for scattering enhancement or attenuation caused by coherent superposition of damage-induced perturbations and the target background; (2) target geometry, inspection partitions, and measurement arrangements are combined to translate the anomalous azimuth and interval width into a preferred observation direction and candidate coverage for the local scanning plane, allowing screening results to directly inform subsequent measurement-region planning; and (3) complex differential imaging, multithreshold analysis, and magnitude-squared-weighted second moments are combined within the candidate region to extract defect locations and equivalent electromagnetic dimensions. These elements establish a multiscale connection from far-field anomaly screening to geometrically constrained scan planning and local quantitative imaging, providing an approach to reducing extensive point-by-point near-field sampling while retaining local spatial characterization capability.

## 2. Methodology

### 2.1. Overall Framework and Electromagnetic Response Mechanism

**Figure 1:**
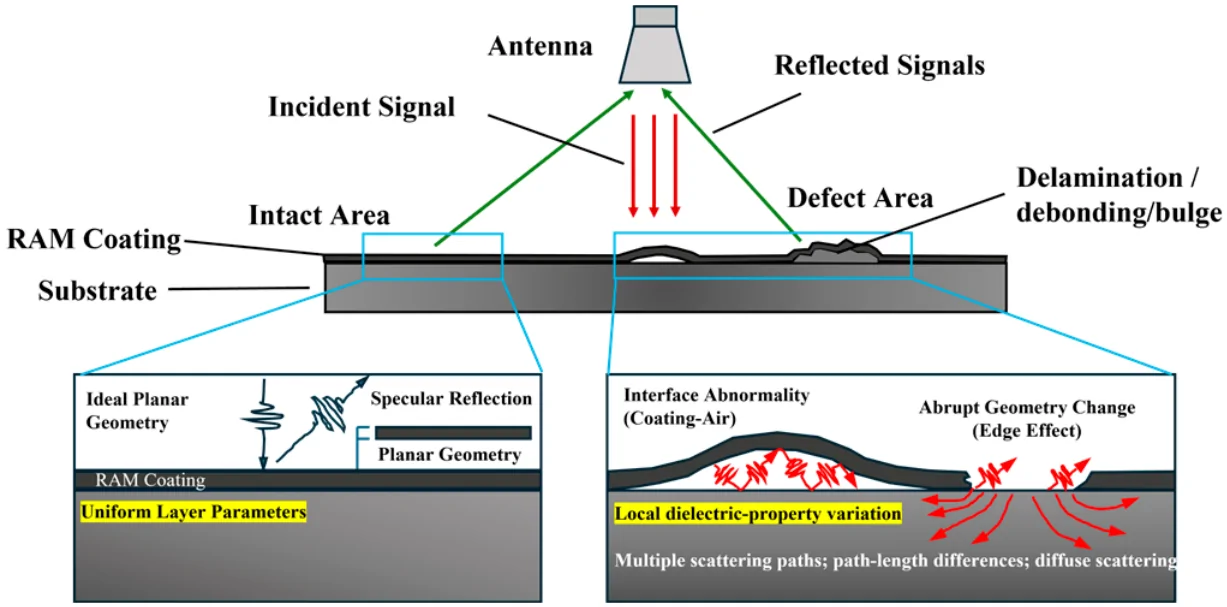
Schematic diagram of electromagnetic response to damage to the absorbing coating.

When the absorbing coating is damaged, the local electromagnetic parameters, boundary conditions, and surface morphology change, thereby disturbing the induced surface current and backscattered echo. Under the same observation conditions, this disturbance manifests as an amplitude-phase difference between the intact state and the damaged state, which can be used for angular domain anomaly screening and local near-field imaging, respectively, as shown in Figure 1. The method compares intact and damaged states. The intact reference can be a baseline recorded when the same object was confirmed intact or an intact object with matched geometry, material, and mounting conditions. Far-field comparisons retain the same frequency, polarization, antenna position, target attitude, and angular reference; near-field complex differencing additionally requires consistent scan coordinates, amplitude scaling, and phase reference. Both states use the same calibration procedure and matched measurement environment to control noncommon responses.

For a fixed frequency and polarization channel, the far-field scattering response of the intact target at azimuth angle *ϕ* is represented by Equation (1).

(1) $$E_{p,0}\left(\phi ;f\right)=\alpha_{p}\left(\phi ;f\right)\int_{\Omega_{t}}J_{p,0}^{\mathrm{eq}}\left(r^{\prime };\phi ,f\right)\exp\left[\mathrm{jk}s\left(\phi \right)\cdot r^{\prime }\right]dA^{\prime }.$$

In the formula, *E_p_*_,0_(*ϕ*; *f*) represents the complex scattering field of the intact state at frequency *f*, polarization channel *p*, and azimuth angle *ϕ*; Ω*_t_* represents the common equivalent reference surface of the target; ***r***′ represents the position vector of the surface integration point; ***s***(*ϕ*) represents the unit vector pointing from the target to the observation direction; *k* = 2*πf*/*c* represents the free space wavenumber; *α_p_*(*ϕ*; *f*) represents the coefficients related to the propagation distance, common propagation factor, and measurement normalization. $J_{p,0}^{\mathrm{eq}}\left(r^{\prime };\phi ,f\right)$ represents the equivalent surface current component under polarization channel *p*, which already includes the surface response generated by the incident field excitation corresponding to the azimuth angle *ϕ* and the received polarization projection. Since this paper uses monostatic angle scanning, the incident direction and the observation direction change synchronously with the azimuth angle, so this equivalent surface current generally also changes with *ϕ*.

When the absorbing coating is damaged, local electromagnetic parameters, surface impedance, or geometry change. The induced surface current under damaged conditions can be expressed as:

(2) $$J_{p,d}^{\mathrm{eq}}=J_{p,0}^{\mathrm{eq}}+\Delta J_{p}^{\mathrm{eq}}.$$

In the equation, $J_{p,d}^{\mathrm{eq}}$ and $J_{p,0}^{\mathrm{eq}}$ are the equivalent surface current components of the damaged state and the intact state, respectively, and $\Delta J_{p}^{\mathrm{eq}}$ is the change in induced surface current caused by damage; substituting Equation (2) into Equation (1), the complex scattering field under the damaged state can be written as:

(3) $$E_{p,d}\left(\phi ;f\right)=\alpha_{p}\left(\phi ;f\right)\int_{\Omega_{t}}\left[J_{p,0}^{\mathrm{eq}}+\Delta J_{p}^{\mathrm{eq}}\right]\exp[jks(\phi )\cdot r^{\prime }]dA^{\prime }.$$

In the equation, *E_p_*_,*d*_(*ϕ*; *f*) represents the complex scattering field of the damaged state at frequency *f*, polarization channel *p*, and azimuth angle *ϕ*. When the same measurement conditions are used for both states, Equations (1) and (3) use the same *α_p_*(*ϕ*; *f*) and equivalent reference surface Ω*_t_*. The difference in complex scattering field between the intact state and the damaged state is defined as:

ΔE*_p_*(*ϕ*; *f*) = *E_p_*_,*d*_(*ϕ*; *f*) − *E_p_*_,0_(*ϕ*; *f*). (4)

When the damage and the resulting major surface response changes are concentrated in the local region Ω*_d_*, Equation (4) can be further expressed as follows:

(5) $${\Delta E}_{p}\left(\phi ;f\right)\approx \alpha_{p}\left(\phi ;f\right)\int_{\Omega_{d}}{\Delta J}_{p}^{\mathrm{eq}}\left(r^{\prime };\phi ,f\right)\exp\left[jks\left(\phi \right)\cdot r^{\prime }\right]dA^{\prime }.$$

Equation (5) shows that the local equivalent surface response changes caused by coating damage will generate an additional complex scattering field, further altering the amplitude and phase responses of the target at different azimuth angles.

At a given frequency, receiving polarization, and observation azimuth, the squared-field angular scattering response for state comparison is defined as:

(6) $$Q_{p}\left(\varphi ;f\right)={\left|E_{p}(\varphi ;f)\right|}^{2}.$$

Here, *E_p_* is the complex scattering-field component for the selected polarization, and |·| denotes complex magnitude. If *E_p_* is expressed in electric-field units, *Q_p_* has the corresponding squared-field units; for a normalized complex response, its units follow the normalization convention. This quantity describes scattering-response strength rather than total energy integrated over time, area, or solid angle.

According to Equation (4), the difference in angular-domain squared-field response between the damaged state and the undamaged state is:

(7) ΔQp=Qp,d−Qp,0=|ΔEp|2+2Re{Ep,0*ΔEp}.

Here, * denotes complex conjugation and Re{·} denotes the real part. The two terms on the right are the perturbation-field self-response and its coherent cross term with the intact background field; the cross term may be positive or negative.

Let *S_mn_*(*f_l_*) denote the complex echo acquired at the l-th frequency point from the (*m*, *n*)-th scan position. The corresponding damaged-state echo is:

*S_mn_*_,*d*_(*f_l_*) = *S_mn_*_,0_(*f_l_*) + Δ*S_mn_*(*f_l_*). (8)

In the formula, *m* and *n* are the discrete position indices in two directions of the scanning plane, *l* is the frequency sampling point index, *f_l_* is the *l*-th frequency point, *S_mn_*_,0_(*f_l_*) is the complex echo acquired at frequency *f_l_* at the (*m*, *n*)-th scanning position in the intact state, *S_mn_*_,*d*_(*f_l_*) is the corresponding complex echo in the damaged state, and Δ*S_mn_*(*f_l_*) is the change in complex echo caused by damage. The measured complex echo contains amplitude and phase information and is response-calibrated using the common references described in Section 3.2. At the same frequency, polarization, and measurement configuration, a common multiplicative transfer factor cancels in the ratio of the two state responses. The angular metric uses the squared magnitude, whereas near-field imaging and differencing retain phase information.

For far-field screening at a fixed frequency *f*_0_ and polarization channel *p*, the intact- and damaged-state squared-field responses are divided by the same positive reference *Q_ref_*. Equations (9) and (10) define the electromagnetic scattering energy ratio (ESER), a dimensionless normalized squared-field response ratio. The quantities *Q_ref_* and *Q_p_* have the same units, and a common reference convention is used for both states and across the compared angular range. Subscripts 0 and *d* denote the intact and damaged states, respectively.

(9) $${\mathrm{ESER}}_{p,0}^{\mathrm{lin}}\left(\phi ;f_{0}\right)=\frac{Q_{p,0}\left(\phi ;f_{0}\right)}{Q_{\mathrm{ref}}\left(f_{0}\right)},$$

(10) $${\mathrm{ESER}}_{p,d}^{\mathrm{lin}}\left(\phi ;f_{0}\right)=\frac{Q_{p,d}\left(\phi ;f_{0}\right)}{Q_{\mathrm{ref}}\left(f_{0}\right)}.$$

The logarithmic form of ESER is defined as:

(11) $${\mathrm{ESER}}_{p,0}^{\mathrm{dB}}(\phi ;f_{0})=10\;\log_{10}\left[{\mathrm{ESER}}_{p,0}^{\mathrm{lin}}\left(\phi ;f_{0}\right)\right],$$

(12) $${\mathrm{ESER}}_{p,d}^{\mathrm{dB}}(\phi ;f_{0})=10\;\log_{10}\left[{\mathrm{ESER}}_{p,d}^{\mathrm{lin}}(\phi ;f_{0})\right].$$

In the formula, ${\mathrm{ESER}}_{p,0}^{\mathrm{dB}}(\phi ;f_{0})$ and ${\mathrm{ESER}}_{p,d}^{\mathrm{dB}}(\phi ;f_{0})$ are the logarithmic ESER for the intact coating state and the damaged state, respectively. The ESER curves in the following text all adopt this logarithmic form.

To characterize the angular domain variation in the damaged state relative to the intact coating reference, the azimuth difference factor is defined as:

(13) $$F_{p}\left(\phi ;f_{0}\right)=10\;\log_{10}\left[\frac{Q_{p,d}(\varphi ;f_{0})}{Q_{p,0}(\varphi ;f_{0})}\right]=20\;\log_{10}\left[\frac{\left|E_{p,d}(\varphi ;\;f_{0})\right|}{\left|E_{p,0}(\varphi ;\;f_{0})\right|}\right].$$

The common positive reference *Q_ref_* cancels in Equation (13). ESER displays the dynamic range of the two state responses on a common reference and logarithmic scale, while *F_p_* extracts their relative change: positive values indicate enhancement and negative values indicate reduction, in dB. The two representations contain the same measurement information and serve state-curve presentation and anomalous-interval extraction, respectively. The logarithmic ratio is evaluated for positive responses at angular samples with adequate signal-to-noise ratio (SNR).

**Figure 2:**
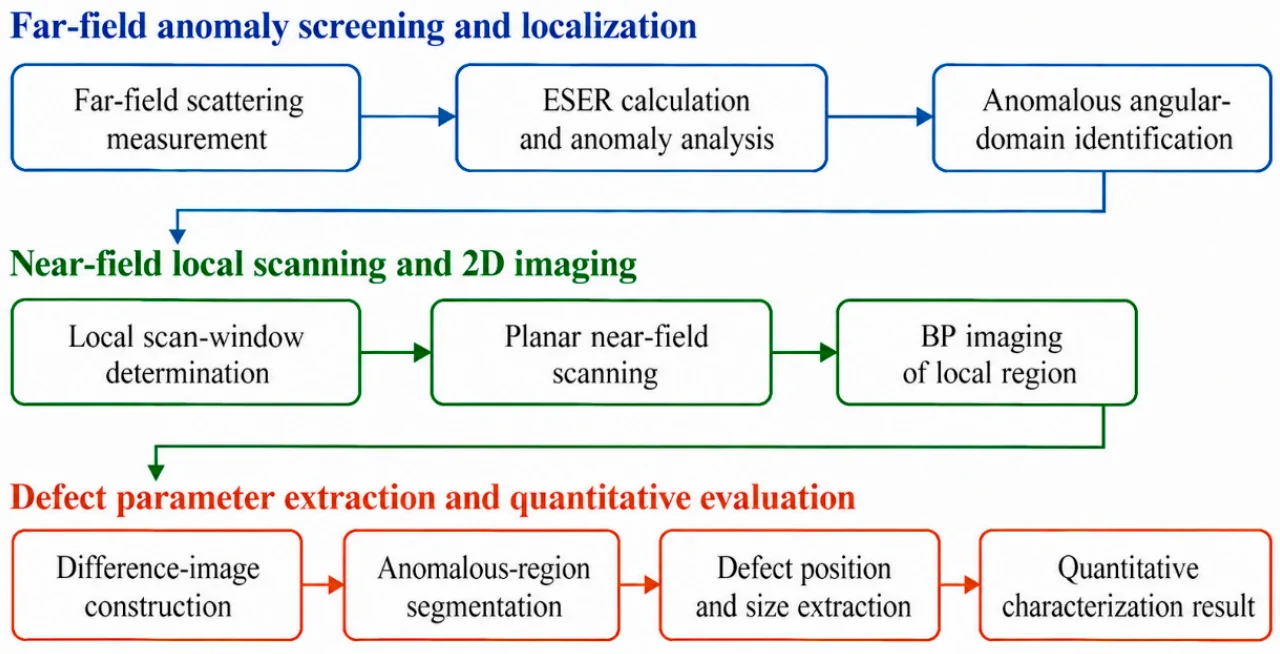
Flowchart of multiscale detection combining far-field angular-domain screening and local near-field imaging.

The overall detection workflow of this paper is shown in Figure 2.

### 2.2. Far-Field Angular-Domain Anomaly Screening

Under fixed analysis frequency, elevation angle, and polarization conditions, calculate the azimuth difference factor *F_p_*(*ϕ*; *f*_0_) for discrete azimuth samples. Considering that damage may cause enhanced or weakened scattering, the anomalous response amplitude is defined as:

*A_p_*(*ϕ_i_*) = ∣*F_p_*(*ϕ_i_*; *f*_0_)∣. (14)

To reduce the impact of manual threshold selection on anomaly interval determination, robust estimation of the angular domain background response is performed using the median and median absolute deviation: *m_p_* = *median_i_A_p_*(*ϕ_i_*), *s_p_* = 1.4826 *median_i_*∣*A_p_*(*ϕ_i_*) − *m_p_*∣, and the anomaly determination threshold is defined as:

*T_p_* = *m_p_* + 3*s_p_*. (15)

When *A_p_*(*ϕ_i_*) > *T_p_*, the corresponding azimuth sample is considered a candidate outlier. To suppress isolated noise and local random fluctuations, only outlier responses containing at least two consecutive azimuth samples are retained, and adjacent outlier samples are merged into valid anomalous azimuth intervals:

(16) $$\Omega_{p}=\overset{K_{p}}{\underset{k=1}{⋃}}\Omega_{p,k},\;\Omega_{p,k}=\left[\phi_{p,k}^{\mathrm{sta}},\phi_{p,k}^{\mathrm{end}}\right].$$

In the formula, *K_p_* represents the number of valid anomaly intervals identified; $\phi_{p,k}^{\mathrm{sta}}$ and $\phi_{p,k}^{\mathrm{end}}$ are the starting and ending azimuth angles of the *k*-th anomaly interval, respectively.

Within the *k*-th anomaly interval, the set of angles reaching the maximum azimuth difference amplitude is first determined, and then the corresponding main anomaly azimuth is determined:

(17) $$\Phi_{p,k}^{\max}=\arg\underset{\phi_{j}\in \Omega_{p,k}}{\max}|F_{p}\left(\phi_{j};f_{0}\right)|,\;\phi_{p,k}^{\mathrm{peak}}=\mathrm{median}\left(\Phi_{p,k}^{\max}\right).$$

In the formula, $\Phi_{p,k}^{\max}$ represents the set of azimuth angle samples that reach the maximum difference amplitude within the interval. When the maximum value is unique, $\phi_{p,k}^{\mathrm{peak}}$ is the azimuth angle corresponding to that maximum value; when the maximum amplitude is attained at multiple azimuth angles, the median of those angles is taken as the principal anomalous azimuth of the interval, thus ensuring the uniqueness of the peak azimuth definition.

The angular domain width of the *k*-th anomalous interval is defined as:

(18) $$\Delta \phi_{p,k}={\tilde{\phi }}_{p,k}^{\mathrm{end}}-{\tilde{\phi }}_{p,k}^{\mathrm{sta}}.$$

Here, $\tilde{\phi }$ denotes the unwrapped azimuth angle. The anomalous intervals and main anomalous azimuths under different polarization modes are extracted independently. To simplify the subsequent expressions, the anomalous interval number k is omitted in Equations (19)–(26).

### 2.3. Mapping from the Angular Anomaly to the Local Scan Window

Detection zones are predefined from target shape, structural boundaries, and measurement accessibility, such as a scannable nose-side region, rather than from known defect coordinates. Within these zones, angular anomalies identify preferred observation directions and guide candidate near-field windows; near-field imaging then determines defect position and equivalent dimensions. Here, “global-to-local” denotes a scan-planning procedure constrained by the target geometry and predefined structural detection zone. Its numerical demonstration is limited to the investigated configurations; application to other aircraft geometries or defect positions requires assessment of the corresponding geometric projection and candidate-window coverage. For far-field measurements, the elevation angle remains constant. The target’s geometric center is set at *O*, and the far-field observation azimuth uses a fixed direction in the target coordinate system as the zero-degree reference direction. The corresponding priority observation direction unit vector is:

(19) $$n_{p}=\left[\begin{array}{c} \cos\phi_{p}^{\mathrm{peak}}\\ \sin\phi_{p}^{\mathrm{peak}}\\ 0 \end{array}\right].$$

Equation (19) gives the horizontal preferred observation direction determined by the principal anomalous azimuth; its third component is zero. This direction and the structural zone jointly define the candidate window: the anomalous interval constrains azimuthal coverage, and the zone geometry provides the center height and height range. The same azimuth can correspond to different heights, so the two-dimensional defect position is subsequently determined from near-field imaging within the candidate region.

Let *d* denote the distance from the target reference center to the center of the geometric projection plane. The center of this plane is then given by:

***C****_p_* = ***O*** + *d**n**_p_*. (20)

Here, *O* is the target reference center, *C_p_* is the center of the geometric projection plane, and *n_p_* is its normal. The distance *d* converts an angular interval into a coverage dimension on the reference plane. The lift-off *h* is the normal separation between the actual scanning plane and the local target reference plane. The antenna-to-target distance in the far-field setup serves a separate measurement purpose.

The azimuthal extent of the scanning plane is further determined by the anomalous angular interval. Assume the main anomaly azimuth is located within the selected continuous anomaly interval, and the angle between the main anomaly azimuth and the directions of both side boundaries is less than 90°, i.e., 0 < $\phi_{p}^{\mathrm{peak}}-\phi_{p}^{\mathrm{sta}}<90{}^{\circ}$, 0 < $\phi_{p}^{\mathrm{end}}-\phi_{p}^{\mathrm{peak}}<90{}^{\circ}$.

When the corresponding preferred observation direction is used as the scanning plane normal, the boundary directions on both sides of the anomaly interval can form finite intersections with the reference scanning plane. The two intersections are located on either side of the main anomaly azimuth reference position, and their azimuth projection distance is:

(21) $$l_{1,p}=\mathrm{dtan}(\phi_{p}^{\mathrm{peak}}-\phi_{p}^{\mathrm{sta}}),$$

(22) $$l_{2,p}=\mathrm{dtan}\left(\phi_{p}^{\mathrm{end}}-\phi_{p}^{\mathrm{peak}}\right).$$

In the formula, *l*_1,*p*_ and *l*_2,*p*_ represent the spatial distances of the anomalous angular domains extending to both sides of the scan center in the azimuth direction of the scan plane. Therefore, the minimum azimuth coverage length of the anomalous angular domains on the scan plane is:

(23) $$L_{p}=l_{1,p}+l_{2,p}=d\left[\tan\left(\phi_{p}^{\mathrm{peak}}-\phi_{p}^{\mathrm{sta}}\right)+\tan\left(\phi_{p}^{\mathrm{end}}-\phi_{p}^{\mathrm{peak}}\right)\right].$$

When the anomaly interval is approximately symmetrical about the orientation of the main anomaly, we have $\phi_{p}^{\mathrm{peak}}-\phi_{p}^{\mathrm{sta}}\approx \phi_{p}^{\mathrm{end}}-\phi_{p}^{\mathrm{peak}}\approx \frac{\Delta \mathrm{\phi p}}{2}$.

Therefore, under the geometry of the ray and the reference plane, the width of the reference window can be approximately written as:

(24) $$L_{p}\approx 2\mathrm{dtan}\left(\frac{\Delta \mathrm{\phi p}}{2}\right).$$

Therefore, for a fixed geometric projection distance *d*, a wider anomalous angular interval produces a greater projected azimuthal width on the reference plane. Thus, the width of the anomalous angular interval can be used to constrain the azimuth scale of the local scanning window.

The one-sided spatial margin *δ* accommodates angular discretization and scanning-position deviations while retaining intact background for local differential imaging. For an angular separation *β* between the anomaly boundary and principal anomalous azimuth, the first-order change in the one-sided projected distance is

Δ*l* ≈ *tan*(*β*)Δ*d* + *d sec*^2^(*β*)Δ*β*. (25)

Here, Δ*β* is expressed in radians. With *d* = 0.35 m, *β* = 10°, and a 0.5° azimuth step, half-step boundary quantization corresponds to approximately 1.57 mm when the peak angle is fixed. The worst-case combination of half-step peak and boundary quantization corresponds to approximately 3.15 mm. Mechanical positioning accuracy, zone boundaries, and background requirements also inform margin selection. The symmetric window follows Equation (26).

*D_p_* = 2[max(*l*_1,*p*_, *l*_2,*p*_) + *δ*]. (26)

In the formula, *D_p_* represents the azimuth candidate coverage scale with the position corresponding to the main anomaly as the reference center. To facilitate near-field measurement setup, this paper adopts a square scanning aperture with a side length of *D_p_*, where the azimuth scale is determined by the anomaly angular domain, and the scanning height and height coverage range are determined by combining the target geometry and the preset detection zone. Thus, the main anomaly azimuth is used to determine the priority observation direction and the azimuth reference position, while the target geometry and measurement distance jointly determine the final position of the scanning window.

The frequencies of the two stages are selected for angular-domain screening and local spatial characterization, respectively. The far-field stage uses a lower operating band to obtain comparable angular-domain responses while accounting for measurement distance, antenna performance, and link SNR. The near-field stage uses a shorter measurement distance and a higher operating frequency, subject to SNR and spatial sampling requirements, to improve cross-range resolution. The different frequencies are therefore suited to the measurement tasks of the respective stages. Frequency points can be selected within the bands permitted by the instrumentation, material response, and measurement conditions, and are kept identical for the intact/damaged comparison within each stage.

Given the principal anomalous azimuth, the anomalous interval, and the target geometry, Equations (19)–(26) describe the geometric projection of the candidate scanning window and do not explicitly contain frequency. The anomalous azimuth and interval used for the projection are determined by the scattering response at the selected frequency. The two stages are connected through common target coordinates and structural partitions: angular-domain screening determines the preferred observation direction and candidate scanning extent, while near-field imaging within this extent yields the defect location and equivalent dimensions. This connection relies on geometric consistency and does not require identical scattering-peak locations or angular-domain curves in the two frequency bands.

### 2.4. Local Planar Near-Field Imaging

**Figure 3:**
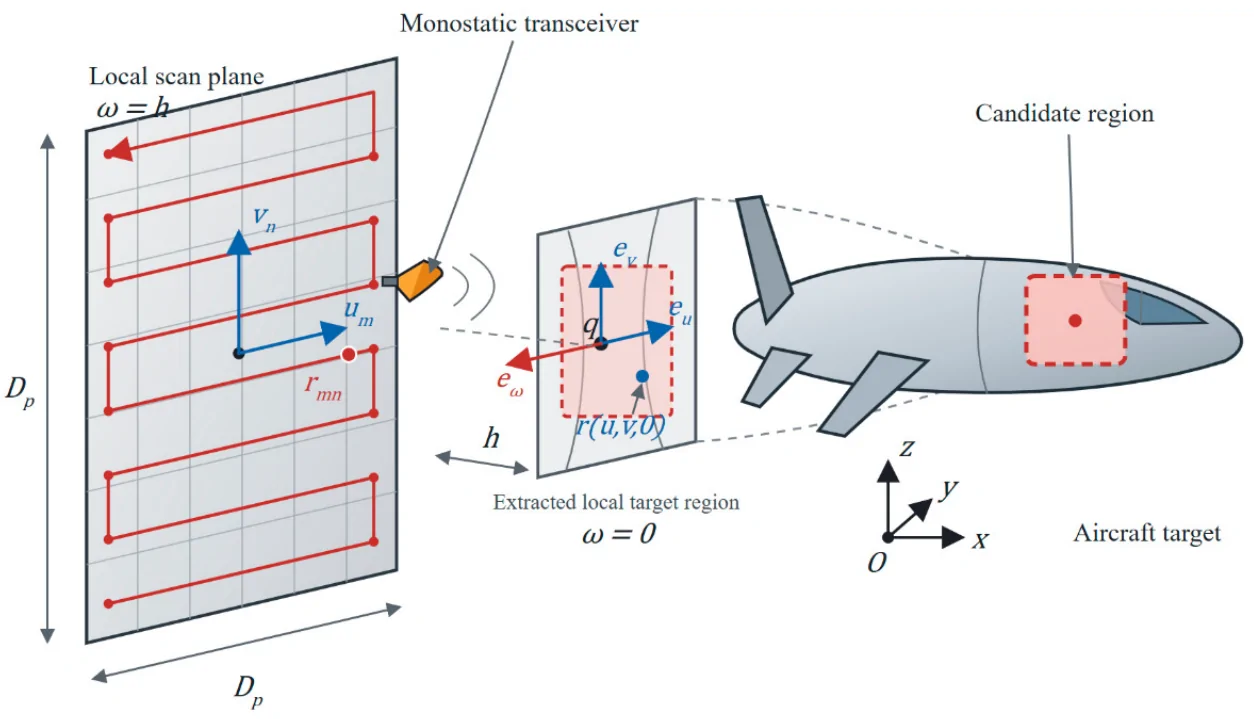
Schematic diagram of planar scanning near-field imaging geometry.

After determining the local scanning plane based on the far-field screening results, a near-field plane scanning method is used to obtain the complex echoes of the candidate region, and local scattering imaging is achieved through coherent back projection. The following analysis focuses on the fixed polarization mode; polarization subscripts are omitted for simplicity. Figure 3 shows a schematic diagram of planar near-field scanning.

Let *q* be the selected target reference point within the candidate region, and *e_u_*, *e_v_* and *e_ω_* be the azimuth, height, and normal unit vectors of the local coordinate system, respectively. The position of any point with local coordinates (*u*, *v*, *ω*) in the target’s global coordinate system is represented as ***r***(*u*, *v*, *ω*) = ***q*** + *u**e**_u_* + *v**e**_v_* + *ω**e**_ω_*. Let the target reference plane be *ω* = 0 and the scanning plane be parallel to this reference plane, with a normal distance of h between them. The (*m*, *n*)th sampling position on the scanning plane is ***r****_mn_* = ***q*** + *u_m_**e**_u_* + *v_n_**e**_v_* + *h**e**_ω_*. Where *u_m_* and *v_n_* are the local coordinates of the scanning position in the azimuth and height directions, respectively. The one-way propagation distance between the scanning position and the imaging point is:

(27) $$R_{\mathrm{mn}}\left(u,v,\omega \right)=∥r-r_{\mathrm{mn}}∥=\sqrt{{\left(u-u_{m}\right)}^{2}+{\left(v-v_{n}\right)}^{2}+{\left(\omega -h\right)}^{2}}.$$

For two-dimensional imaging on the target reference plane *ω* = 0, Equation (27) becomes: $R_{\mathrm{mn}}\left(u,v\right)=\sqrt{{\left(u-u_{m}\right)}^{2}+{\left(v-v_{n}\right)}^{2}+h^{2}}$, where *h* is the normal distance from the antenna scanning plane to the local target reference plane; these planes are *ω* = *h* and *ω* = 0 in the local coordinate system, respectively.

The antenna uses a monostatic transceiver mode. The electromagnetic wave propagates from the antenna to the target and then returns to the receiver, so its propagation phase is determined by the two-way distance. Let the *l*-th frequency be *f_l_*, and the corresponding wave number be $k_{l}=\frac{2\pi f_{l}}{c}$, where c is the electromagnetic wave propagation speed. At the selected frequency and within the local scanning region, approximately common amplitude factors are absorbed into the effective scattering coefficient, yielding the monostatic complex echo in Equation (28).

(28) $$S_{\mathrm{mn}}\left(f_{l}\right)=\int_{\Omega }\rho \left(r\right)\exp\left[-j2k_{l}R_{\mathrm{mn}}\left(r\right)\right]dr.$$

Here, *S_mn_* is the echo at a scanning position, *ρ* is the effective scattering coefficient for the measurement configuration, and Ω is the local imaging region. This approximation requires sufficiently gradual local variations in directivity and coupling, with consistent antenna configuration, polarization, and distance reference between states. Finite aperture and phase-center position affect focusing and equivalent dimensions through the antenna spatial response and distance reference.

Back projection imaging compensates for the two-way propagation phase in Equation (28) based on the propagation distance between the candidate imaging point and each scanning position. For the imaging position *r*, the phase-compensated echo is:

(29) $${\tilde{S}}_{\mathrm{mn}}\left(r,f_{l}\right)=S_{\mathrm{mn}}\left(f_{l}\right)\exp\left[j2k_{l}R_{\mathrm{mn}}\left(r\right)\right].$$

In the formula, ${\tilde{S}}_{\mathrm{mn}}(r,f_{l})$ represents the result of propagation phase compensation of the complex echo at the (*m*, *n*)th scan position for the candidate position *r*.

Subsequently, the compensation results at each scan position are coherently accumulated to obtain the local imaging result:

(30) $$I\left(u,v;f_{l}\right)=\sum_{m=1}^{N_{u}}\sum_{n=1}^{N_{v}}S_{\mathrm{mn}}(f_{l})\exp[j2k_{l}R_{\mathrm{mn}}(u,v)].$$

In the formula, *I*(*u*, *v*; *f_l_*) represents the complex imaging result at the candidate point (*u*, *v*) at the working frequency *f_l_*, and *N_u_* and *N_v_* are the number of sampling points in the azimuth and height directions of the scanning plane, respectively. Equation (30) achieves coherent accumulation of echoes from the same scattering position through two-way propagation phase compensation. At a fixed operating frequency, the complex image is abbreviated as *I*(*u*, *v*), and its magnitude is used to display the local image amplitude.

Curved-surface imaging results are defined in the local reference plane of the candidate region and related to the physical structure through known target geometry. For a local circular section with curvature radius *R* and arc length *s* from the tangency point, the tangential projection *x* and normal deviation *z* satisfy:

(31) $$x=\mathrm{Rsin}\left(\frac{s}{R}\right),z=R\left[1-\cos\left(\frac{s}{R}\right)\right]\approx \frac{s^{2}}{2R}.$$

For small $\frac{\left|s\right|}{R}$, the tangential projection and arc length agree to first order, while the normal deviation is approximately $\frac{s^{2}}{2R}$. This relation supports assessment of the local planar approximation and propagation-path variation. The reported positions and equivalent dimensions refer to the reference plane; interpretation in terms of surface arc length or larger-curvature regions requires the actual structural geometry.

### 2.5. Defect Parameter Extraction and Quantitative Characterization

To highlight the damage response, background cancelation was performed on the complex imaging results of both intact and damaged data under the same scanning grid, amplitude scale, phase reference, and imaging parameters:

Δ*I*(*u*,*v*) = *I_d_*(*u*, *v*) − *I*_0_(*u*, *v*). (32)

Let **x** = (*u*, *v*)*^T^* denote the local image coordinates. For a damaged-state image observed as (1 + *ε_a_*)exp(*jε_φ_*)*I_d_*(**x** + *δ***x**), the first-order difference from the intact reference is:

Δ*I_obs_*(*x*) ≈ Δ*I_d_*(*x*) + *ε_a_I_d_*(*x*) + *jε_φ_I_d_*(*x*) + ∇*_x_I_d_*(*x*)·*δx*. (33)

Here, *I_d_* is the ideally registered damaged-state complex image, *δx* is a small displacement vector in the local imaging plane, ∇*_x_* is the gradient with respect to (*u*, *v*), *ε_a_* is the relative amplitude deviation, and *ε_φ_* is the phase deviation in radians. The ideally registered difference is Δ*I_d_*(*x*) = *I_d_*(*x*) − *I*_0_(*x*). Strong background responses, phase deviations, and spatial gradients influence the residual difference; reference matching should keep these residuals below the defect response to be identified.

The magnitude of the complex differential image is then normalized as follows:

(34) $$A_{\max}=\max\left|\Delta I\left(u,v\right)\right|,G\left(u,v\right)=\frac{\left|\Delta I\left(u,v\right)\right|}{\max\left(A_{\max},\varepsilon \right)},$$

where *G*(*u*, *v*) represents the normalized defect difference map, and *ε* > 0 is a numerical stability constant used to avoid a zero denominator.

#### 2.5.1. Defect Location Extraction

A binary mask is formed at each threshold of the normalized differential image, and connected components are labeled using a fixed pixel-adjacency rule. The component containing the maximum-response point is selected as the main region *B_i_*; tied peaks are resolved using a predefined order consistent across all cases. The squared-magnitude-weighted centroid, second moments and segmented area are calculated only within *B_i_* using Equations (35)–(38). This rule excludes sidelobes and background regions disconnected from the main peak; the effect of responses connected to the main region is assessed through the multi-threshold analysis.

(35) $$u_{i}=\frac{\sum_{\left(u,v\right)\in B_{i}}uG^{2}\left(u,v\right)}{\sum_{\left(u,v\right)\in B_{i}}G^{2}\left(u,v\right)},v_{i}=\frac{\sum_{\left(u,v\right)\in B_{i}}vG^{2}\left(u,v\right)}{\sum_{\left(u,v\right)\in B_{i}}G^{2}\left(u,v\right)}.$$

In the formula, (*u_i_*, *v_i_*) represents the squared-magnitude-weighted centroid of the differential image of the main anomaly region in the local imaging plane under the *i*-th threshold. Here, *u_i_* represents the azimuth position, and *v_i_* represents the height position.

#### 2.5.2. Defect Size Extraction

For each candidate threshold, the central second-moment matrix of the main connected component, weighted by the squared magnitude of the differential image, is calculated using Equation (36). The corresponding equivalent electromagnetic length and width are obtained from Equation (37). The central second-moment matrix of the *i*-th main region is:

(36) $$M_{i}=\frac{\sum_{(u,v)\in B_{i}}G^{2}\left(u,v\right)\left[\begin{array}{cc} {\left(u-u_{i}\right)}^{2} & \left(u-u_{i})(v-v_{i}\right)\\ \left(u-u_{i})(v-v_{i}\right) & {\left(v-v_{i}\right)}^{2} \end{array}\right]}{\sum_{(u,v)\in B_{i}}G^{2}\left(u,v\right)}.$$

Let *λ_i_*_,1_ and *λ_i_*_,2_ be the eigenvalues of ***M****_i_*, with *λ_i_*_,1_ ≥ *λ_i_*_,2_. The corresponding equivalent length and width are:

(37) $$L_{i}=\sqrt{12\lambda_{i,1}},W_{i}=\sqrt{12\lambda_{i,2}}.$$

Equation (37) converts the squared-magnitude differential response into the dimensions of a rectangle with the same principal second moments, using the central second-moment relation for a uniform rectangle. These quantities are termed the equivalent electromagnetic length and width and characterize the spatial extent of the defect-related response in the reference imaging plane. Their relationship to the physical defect boundaries depends on the scattering distribution; the two are therefore not directly interchangeable.

The segmented area of the main differential-response region is determined by its pixel count:

*A_i_* = *N_i_*Δ*u*Δ*v*. (38)

In the formula, *N_i_* represents the number of pixels in region *B_i_*, and Δ*u* and Δ*v* are the pixel intervals in the two imaging directions, respectively.

The centroid and equivalent dimensions of the main region are calculated over a predefined increasing threshold grid:

(39) $$J=\frac{s_{L}}{\bar{L}}+\frac{s_{W}}{\bar{W}}.$$

Here, $\bar{L}$ and $\bar{W}$ are the mean values of *L_i_* and *W_i_* within a candidate threshold interval, and *s_L_* and *s_W_* are their corresponding standard deviations over the same interval. Thus, *J* is a dimensionless measure of the relative variation in the equivalent length and width. Candidate stable intervals contain the same number of consecutive threshold values; only intervals with nonempty main regions and positive mean dimensions are retained. *J* is calculated using Equation (39), and the interval with the smallest value is selected, with ties resolved using a fixed ordering rule. Final parameters are the medians within that interval, as specified in Equation (40). *J* measures the threshold sensitivity of length and width only; centroid variation is assessed separately from *u_i_* and *v_i_* across thresholds.

The final defect location and size are both taken as the median of the threshold calculation results within the stable interval:

*u_c_* = *med*(*u_i_*), *v_c_* = *med*(*v_i_*), *L* = *med*(*L_i_*), *W* = *med*(*W_i_*), *A* = *med*(*A_i_*), (40)

where (*u_c_*, *v_c_*) is the median of the threshold-dependent centroid coordinates and is used as the defect-location estimate, *L* and *W* are the equivalent electromagnetic length and width, and *A* is the segmented area of the main response region.

The extracted center is mapped from the local reference coordinates to the target coordinate system as:

*r_c_* = *q* + *u_c_e_u_* + *v_c_e_v_*, (41)

where *q* is the local target reference point, and *e_u_* and *e_v_* are the unit vectors in the local azimuth and height directions, respectively.

For the simulation model, the true location and geometric dimensions of the defect are known; therefore, the parameter extraction results can be further evaluated. The positioning error is defined as:

(42) $$e_{\mathrm{pos}}=\sqrt{{\left(u_{c}-u_{0}\right)}^{2}+{\left(v_{c}-v_{0}\right)}^{2}}.$$

Here, (*u*_0_, *v*_0_) denotes the known defect-center coordinates in the same local reference plane and coordinate system as the extracted center (*u_c_*, *v_c_*). The quantity *e*_pos_ is therefore an in-plane localization error. Equation (43) gives the percentage deviation of each extracted quantity from its specified reference. The nominal side lengths are used for rectangular coating loss, whereas a nominal geometric feature scale of 10 mm is used for the impact and bulge cases. *L*_0_, *W*_0_ and *A*_0_ denote the selected length, width and area references, respectively; the segmented area is calculated from the main-region pixel count in Equation (38). Equations (42) and (43) define position error, equivalent-scale deviation and segmented-area deviation as separate quantities. The results below report extracted center coordinates for the numerical cases and equivalent length and width deviations for the numerical and experimental cases.

(43) $$e_{L}=\frac{|L-L_{0}|}{L_{0}}\times 100\%,e_{W}=\frac{|W-W_{0}|}{W_{0}}\times 100\%,e_{A}=\frac{|A-A_{0}|}{A_{0}}\times 100\%.$$

## 3. Results

This section evaluates the method through numerical analysis and a physical experiment. A simplified aircraft-like model from a public source [21] is used to examine angular-domain screening and near-field characterization of impact, coating loss and bulging. A generic coated conical specimen is used to test the measurement workflow for localized coating loss. The numerical results examine the electromagnetic responses of different perturbations, while the experiment demonstrates the integration of the two stages and equivalent-scale extraction. The models and associated data used in this study do not involve confidential aircraft design specifications, sensitive or classified information, or restricted engineering data.

### 3.1. Simulation Verification

**Figure 4:**
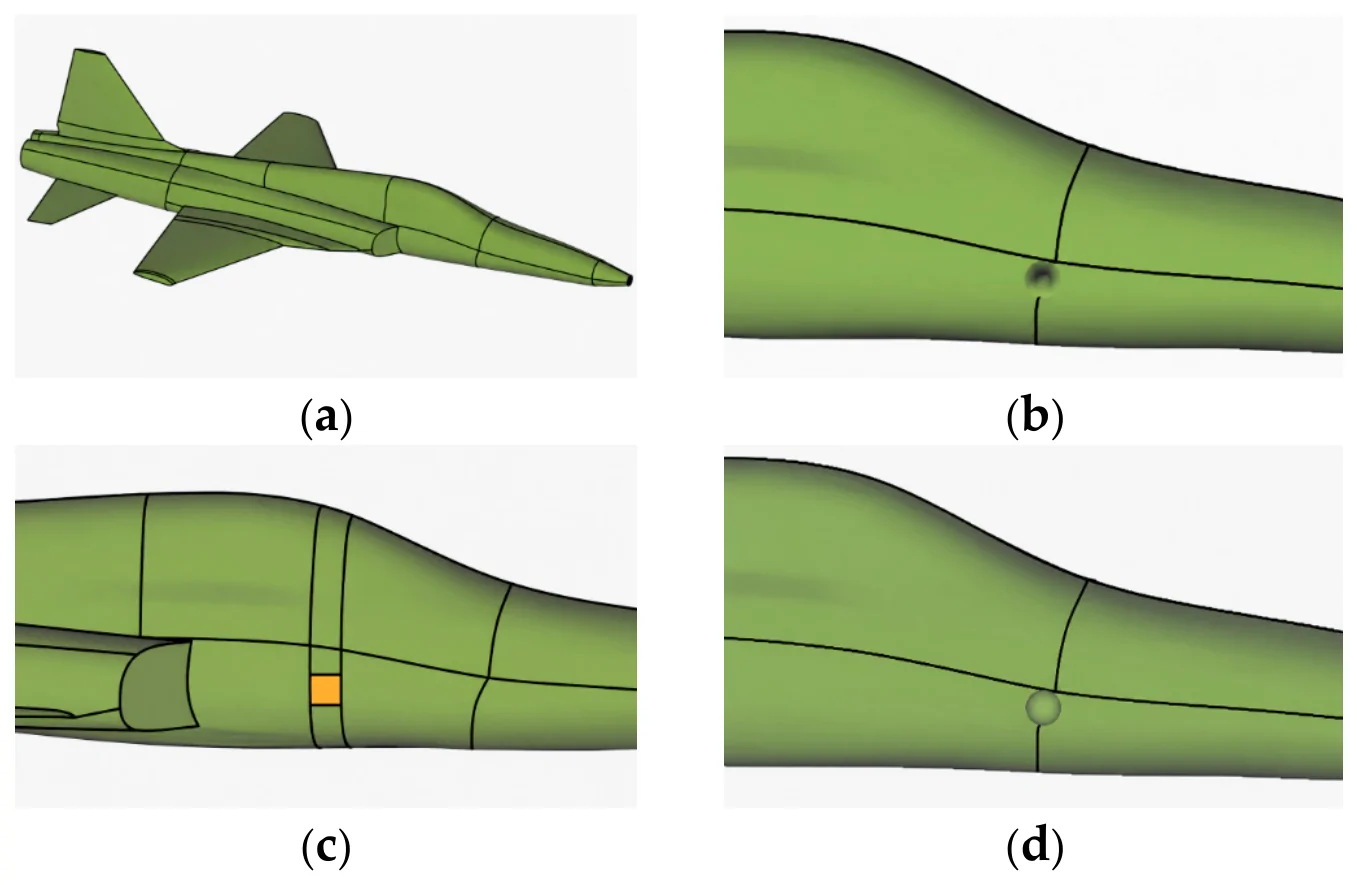
Aircraft model and representative defects: (**a**) overview of the aircraft model; (**b**) impact defect; (**c**) coating-loss defect; (**d**) bulging defect.

For the aircraft model, this paper sets three representative defects: impact defects, bulging defects and coating loss. For impact defects and bulging, a semi-circular defect with a radius of 5 mm is set on the right side of the aircraft nose; for coating loss, a rectangular defect with a size of 10 mm × 10 mm is set. The constructed aircraft model with damage is shown in Figure 4.

The scaled-down aircraft model has a fuselage length of approximately 0.7 m and a wingspan of approximately 0.4 m. The aircraft skin substrate is modeled as a perfect electric conductor (PEC) and is uniformly coated with a 0.2 mm thick layer of absorbing material. In FEKO, the absorbing material uses a frequency-independent medium model, with a relative permittivity set to *ε_r_* = 3.4 and a conductivity set to σ = 50 S/m. In Figure 4, the green area represents the intact absorbing material layer. Damaged areas are simulated by removing parts of the absorbing material layer, exposing the underlying PEC metal substrate to characterize the local failure state of the absorbing coating.

**Figure 5:**
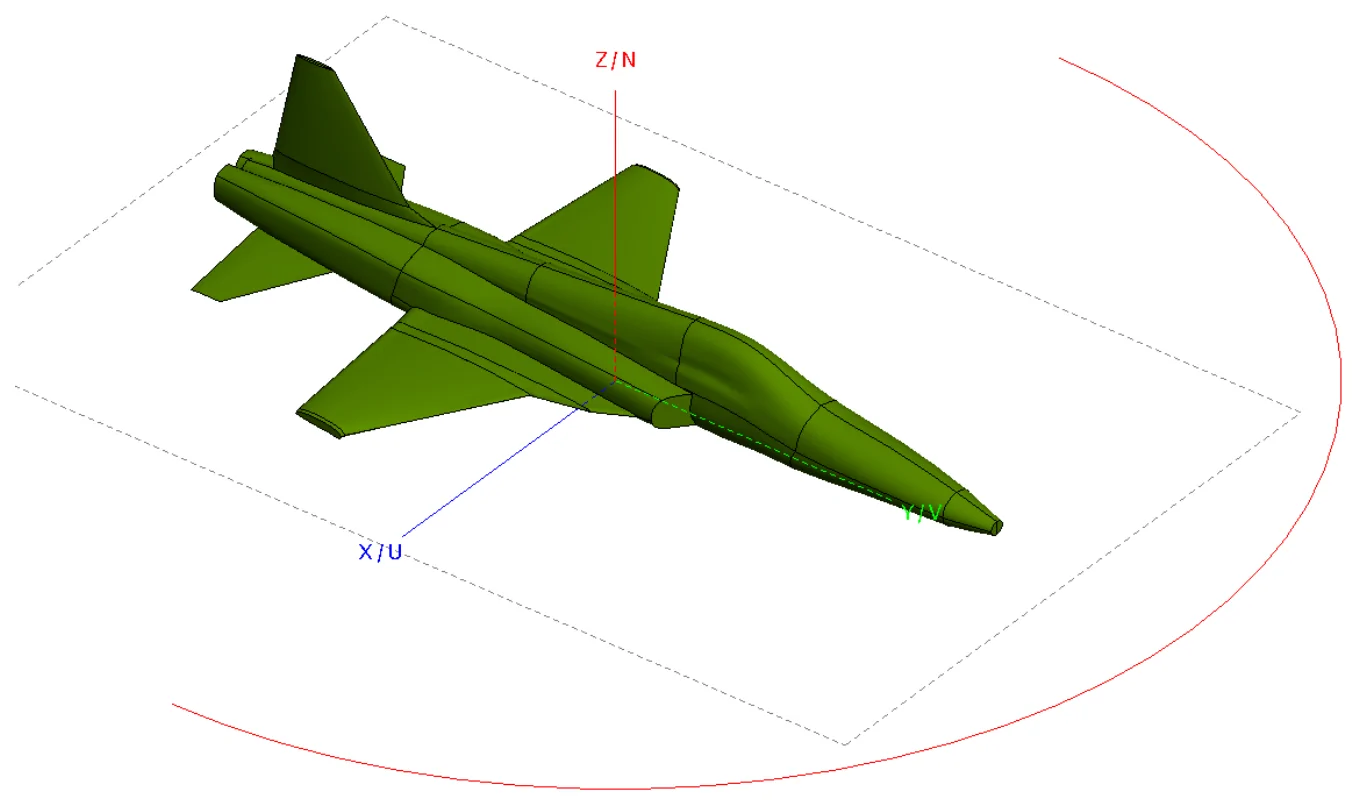
Schematic diagram of far-field scanning of the aircraft model.

In the far-field initial screening stage, this paper first acquires far-field scattering data from the aircraft model. The simulation frequency is set to 12 GHz, and a monostatic far-field observation method is used. The target is scanned under nose-facing incidence conditions (θ = 90°). The scanning angular domain is 180°, the angular step size is 0.5°, and a total of 361 angular sampling points are obtained. The far-field scan of the aircraft is shown in Figure 5.

Angular scattering data were acquired under the same operating conditions for four types of aircraft models: the intact reference model and models with impact defects, bulges, and coating-loss defects. The azimuth difference factor relative to the complete intact reference model was calculated for each damaged model, and near-field imaging and parameter extraction were performed on the corresponding local regions.

#### 3.1.1. Impact Defects

**Figure 6:**
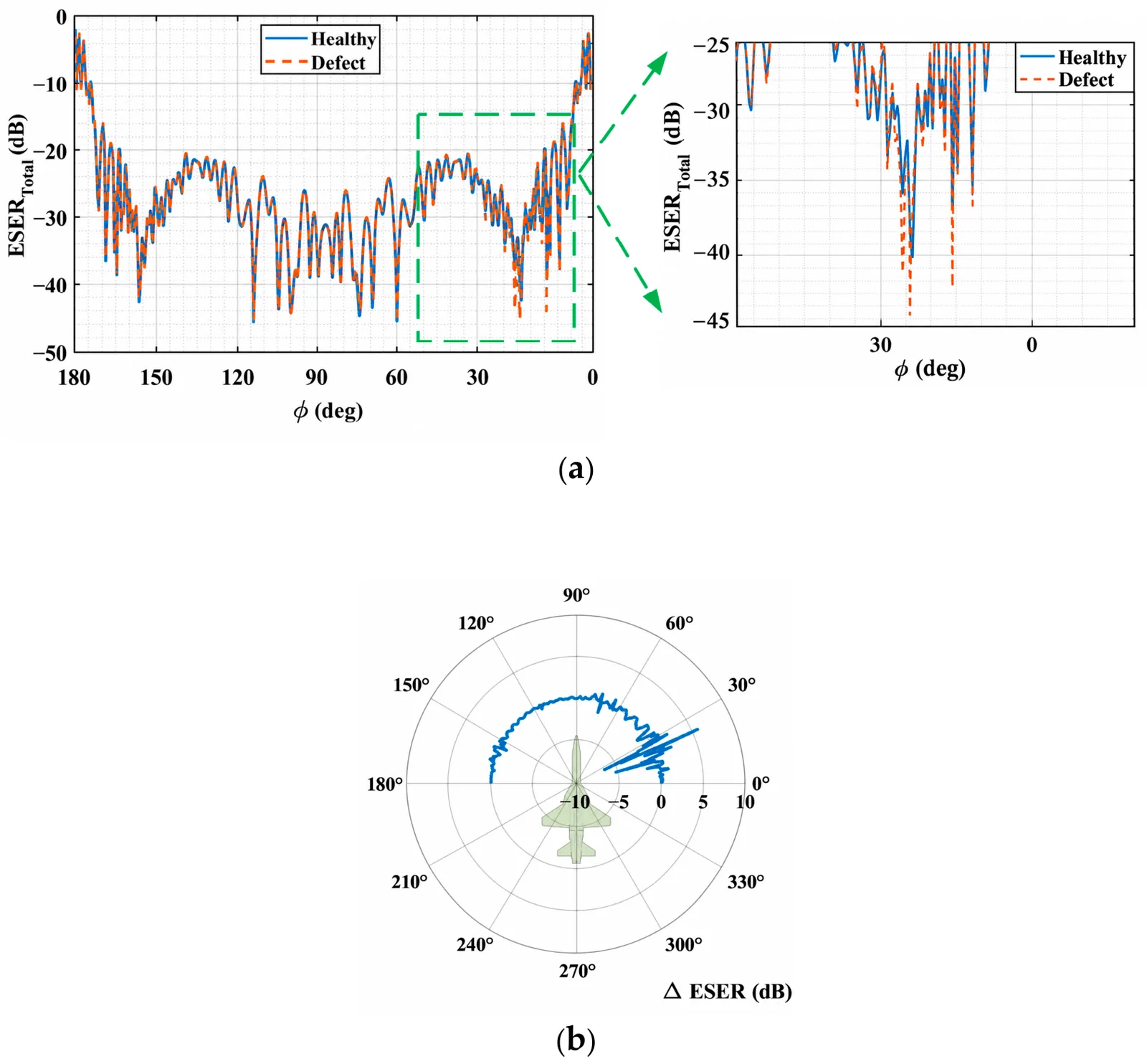
Far-field screening results for the aircraft model with an impact defect: (**a**) logarithmic ESER for the intact and damaged states; (**b**) azimuth difference factor relative to the intact reference. The enlarged view in (**a**) shows the region outlined in green, using the same azimuth and response units as the main plot.

The far-field data of the aircraft with impact defects were obtained according to the above far-field scanning settings, and the corresponding ESER was calculated. The results are shown in Figure 6.

According to the method in Section 2.2, from the azimuth difference factor results in Figure 6, we know that $\phi_{p}^{\mathrm{peak}}\approx 22{}^{\circ},\Omega_{V}=[12{}^{\circ},32{}^{\circ}]$, $\Delta \phi_{V}=\phi_{V}^{\mathrm{end}}-\phi_{V}^{\mathrm{sta}}=20{}^{\circ}$.

According to Equation (19), the normal of the scanning plane corresponding to the main anomaly azimuth is $n_{V}=\left[\begin{array}{c} \cos22{}^{\circ}\\ \sin22{}^{\circ}\\ 0 \end{array}\right]$. Therefore, the local scanning surface should be arranged along the anomaly direction of approximately 22°, facing the side of the target aircraft nose. The scanning range is determined using the width of the anomaly angular domain. Using a geometric projection distance of *d* = 0.35 m for window planning, since the anomaly interval is approximately symmetrical about the main anomaly azimuth, according to Equation (24), its corresponding minimum spatial coverage length is *L_p_* = 2 × 0.35tan(10°) = 0.123 m.

**Figure 7:**
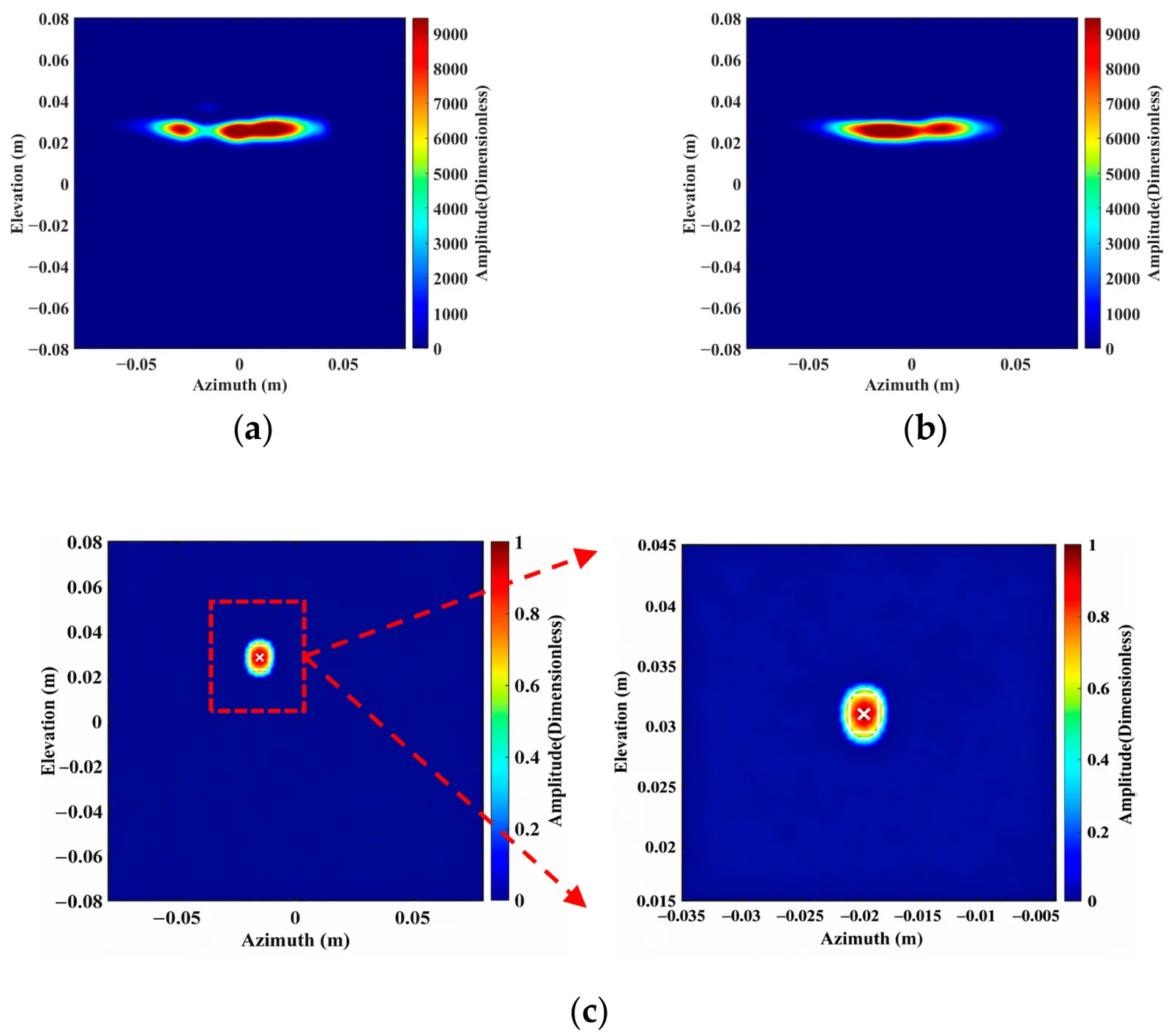
Local near-field imaging and parameter extraction for the aircraft model with an impact defect: (**a**) image of the damaged state; (**b**) image of the intact state; (**c**) normalized differential-response map and an enlarged view of the selected region. Both views in (**c**) use the same local imaging coordinates and normalization; the cross marks the extracted centroid.

A local two-dimensional scan of the aircraft is performed. The two-dimensional imaging result and parameter extraction results are shown in Figure 7.

For the impact case, the extracted center is *v_c_* = 0.028 m and *u_c_* = −0.015 m. The equivalent electromagnetic length and width are 1.093 cm and 0.926 cm, with deviations of 9.3% and 7.4%, respectively, from the nominal geometric feature scale of 10 mm.

#### 3.1.2. Coating Loss

**Figure 8:**
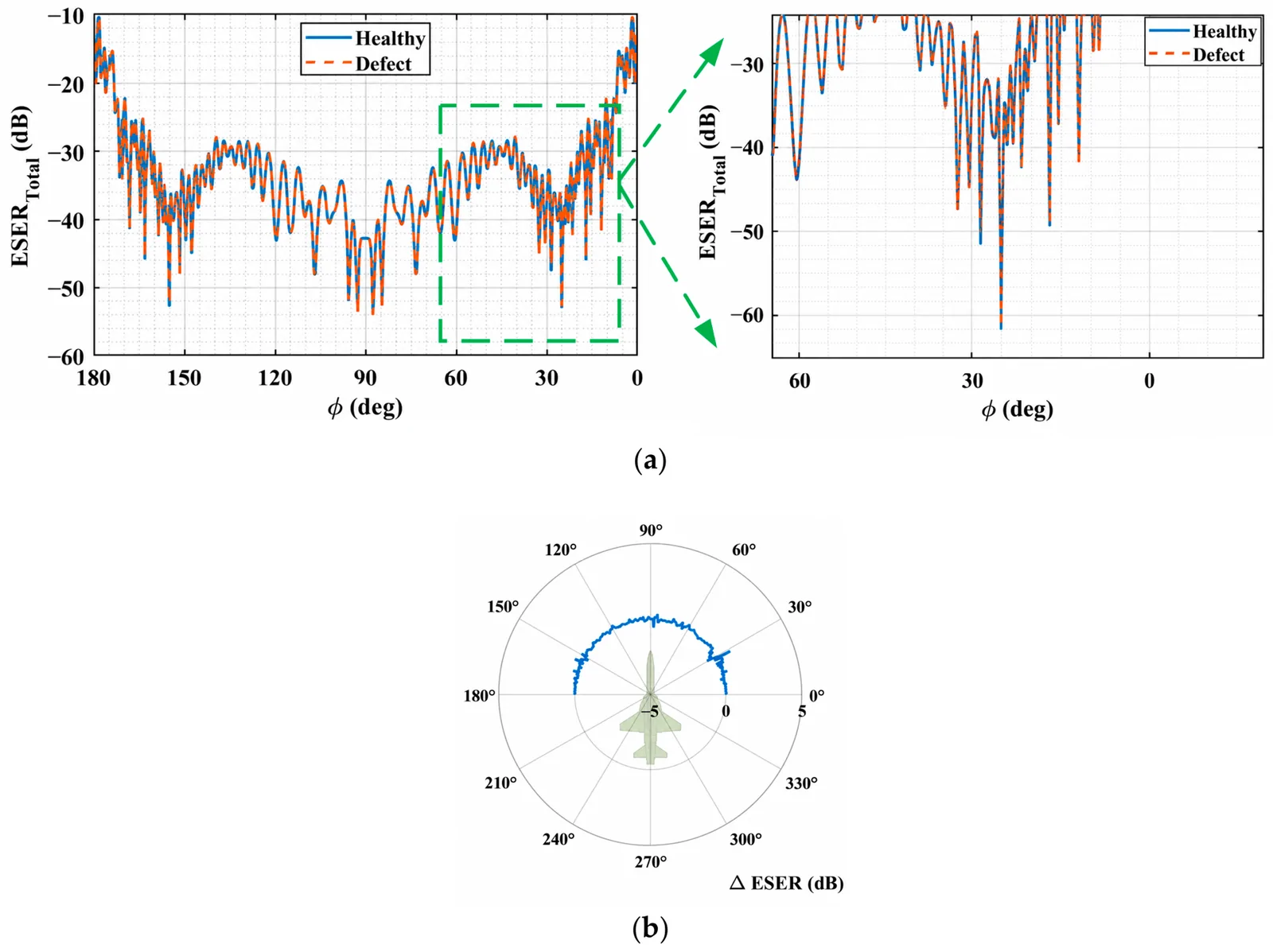
Far-field screening results for the aircraft model with a coating-loss defect: (**a**) logarithmic ESER for the intact and damaged states; (**b**) azimuth difference factor relative to the intact reference. The enlarged view in (**a**) shows the region outlined in green, using the same azimuth and response units as the main plot.

The ESER results for the aircraft model with a coating-loss defect are shown in Figure 8.

According to the method in Section 2.2, from the azimuth difference factor results in Figure 8, we know that $\phi_{p}^{\mathrm{peak}}\approx 24{}^{\circ},\Omega_{V}=[15{}^{\circ},33{}^{\circ}]$, $\Delta \phi_{V}=\phi_{V}^{\mathrm{end}}-\phi_{V}^{\mathrm{sta}}=18{}^{\circ}$.

According to Equation (19), the normal of the scanning plane corresponding to the main anomaly azimuth is $n_{V}=\left[\begin{array}{c} \cos24{}^{\circ}\\ \sin24{}^{\circ}\\ 0 \end{array}\right]$. Therefore, the local scanning plane should be arranged along the anomaly direction of approximately 24°, facing the side of the target aircraft nose. The scanning range is determined using the width of the anomaly angular domain. Using a geometric projection distance of *d* = 0.35 m for window planning, since the anomaly interval is approximately symmetrical about the main anomaly azimuth, according to Equation (24), its corresponding minimum spatial coverage length is *L_p_* = 2 × 0.35 tan (9°) = 0.111 m.

**Figure 9:**
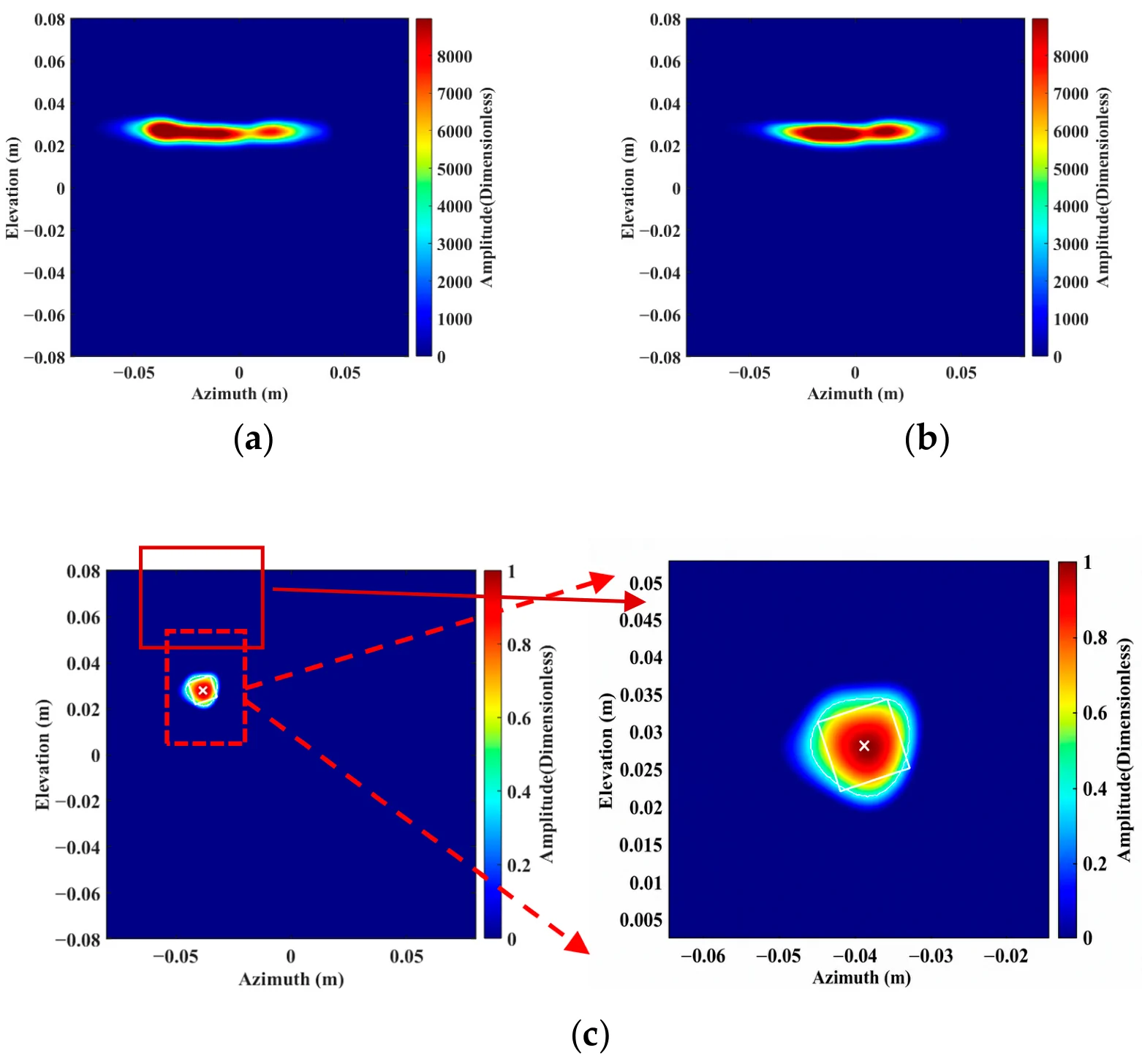
Local near-field imaging and parameter extraction for coating loss on the aircraft model: (**a**) image of the damaged state; (**b**) image of the intact state; (**c**) normalized differential-response map and an enlarged view of the selected region. The enlarged view retains the original local imaging coordinates.

The two-dimensional imaging results and parameter extraction results are shown in Figure 9.

For the rectangular coating-loss case, the extracted center is *v_c_* = 0.028 m and *u_c_* = −0.038 m. The equivalent electromagnetic length and width are 1.086 cm and 0.977 cm, with deviations of 8.6% and 2.3%, respectively, from the nominal side lengths of 10 mm × 10 mm.

#### 3.1.3. Bulging Defect

**Figure 10:**
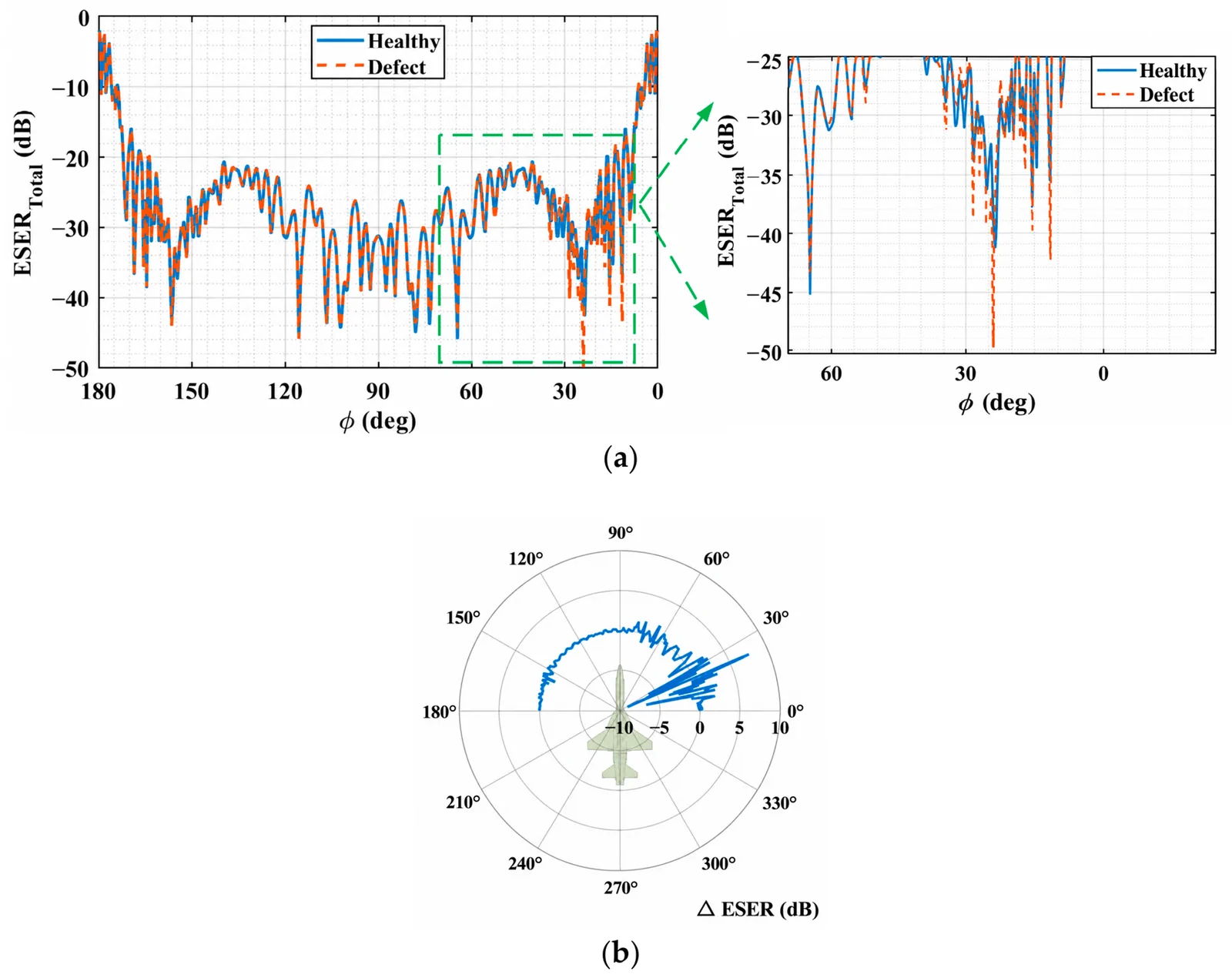
Far-field screening results for the aircraft model with a bulging defect: (**a**) logarithmic ESER for the intact and damaged states; (**b**) azimuth difference factor relative to the intact reference. The enlarged view in (**a**) shows the region outlined in green, using the same azimuth and response units as the main plot.

Figure 10 shows the results obtained from calculating the corresponding ESER of the far-field data of the aircraft with bulge defects.

According to the method in Section 2.2, the azimuth difference factor results in Figure 10 show that $\phi_{p}^{\mathrm{peak}}\approx 18{}^{\circ},\Omega_{V}=[8{}^{\circ},28{}^{\circ}]$, $\Delta \phi_{V}=\phi_{V}^{\mathrm{end}}-\phi_{V}^{\mathrm{sta}}=20{}^{\circ}$.

According to Equation (19), the normal of the scanning plane corresponding to the main anomaly azimuth is $n_{V}=\left[\begin{array}{c} \cos18{}^{\circ}\\ \sin18{}^{\circ}\\ 0 \end{array}\right]$. Therefore, the local scanning plane should be arranged along the anomaly direction of approximately 18°, facing the side of the target aircraft nose. The scanning range is determined using the width of the anomaly angular domain. Using a geometric projection distance of *d* = 0.35 m for window planning, since the anomaly interval is approximately symmetrical about the main anomaly azimuth, according to Equation (24), its corresponding minimum spatial coverage length is L_p_ = 2 × 0.35 tan (10°) = 0.123 m.

**Figure 11:**
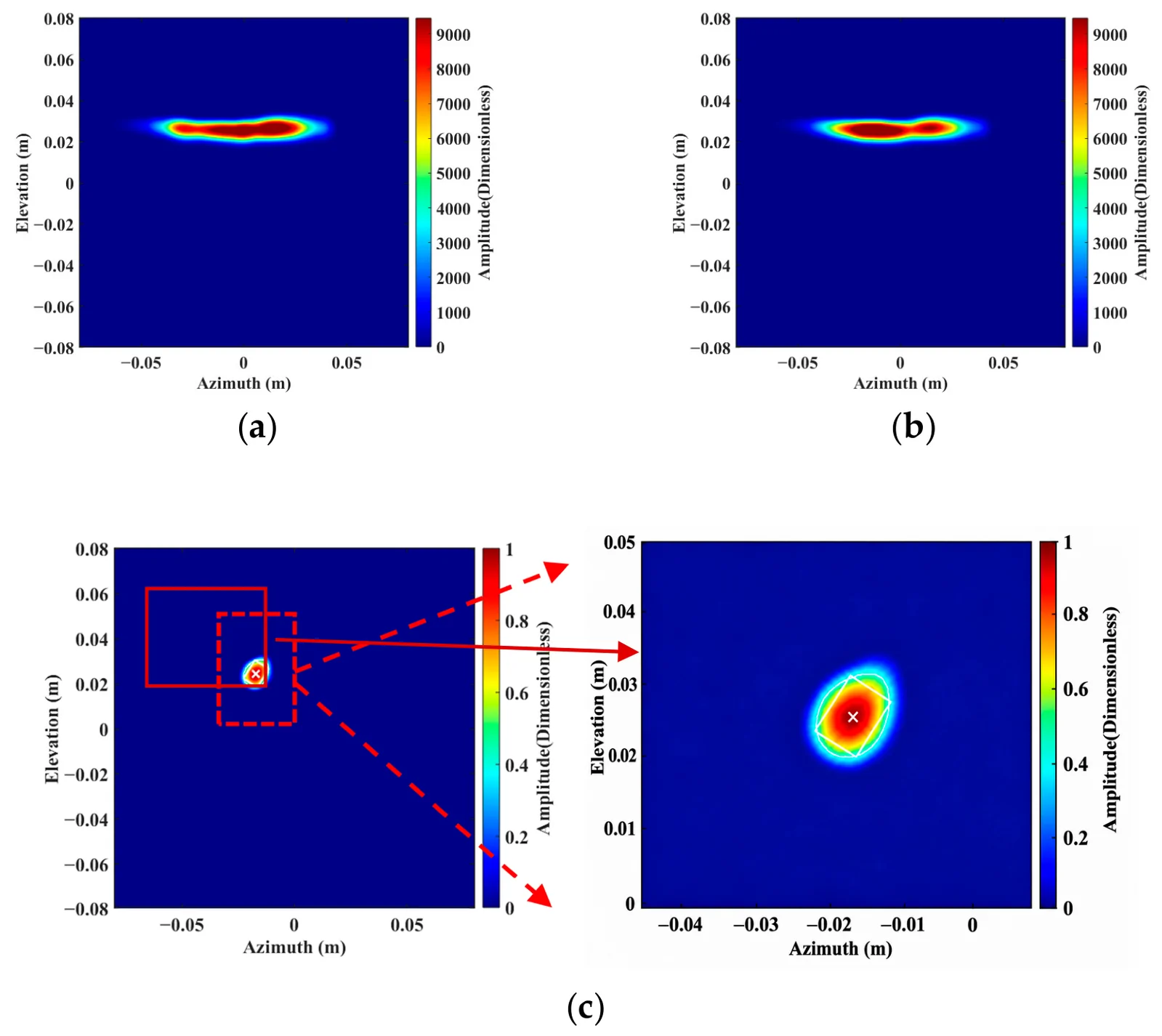
Local near-field imaging and parameter extraction for the aircraft model with a bulging defect: (**a**) image of the damaged state; (**b**) image of the intact state; (**c**) normalized differential-response map and an enlarged view of the selected region. The enlarged view retains the original local imaging coordinates.

The two-dimensional imaging results are shown in Figure 11.

For the bulge case, the extracted center is *v_c_* = 0.025 m and *u_c_* = −0.017 m. The equivalent electromagnetic length and width are 0.965 cm and 0.934 cm, with deviations of 3.5% and 6.6%, respectively, from the nominal geometric feature scale of 10 mm.

Table 1 lists the near-field scanning parameters of the three simulation cases. The principal anomalous azimuths for impact, coating loss, and bulging are 22°, 24°, and 18°, with intervals of 12–32°, 15–33°, and 8–28°, respectively. At a geometric mapping distance of 0.35 m, Equation (24) gives projected widths of 123.43, 110.87, and 123.43 mm. All three cases use a 160 mm × 160 mm scanning window, giving equivalent one-sided margins of 18.29, 24.57, and 18.29 mm relative to these symmetric projected intervals. The centers extracted from the near-field images are (−15, 28), (−38, 28), and (−17, 25) mm in (u, v) coordinates; the corresponding equivalent dimensions are given in Table 2. These values relate candidate-window geometry to the local extraction results, with defect coordinates determined by near-field imaging.

The simulation results are summarized in Table 2.

### 3.2. Experimental Verification

**Figure 12:**
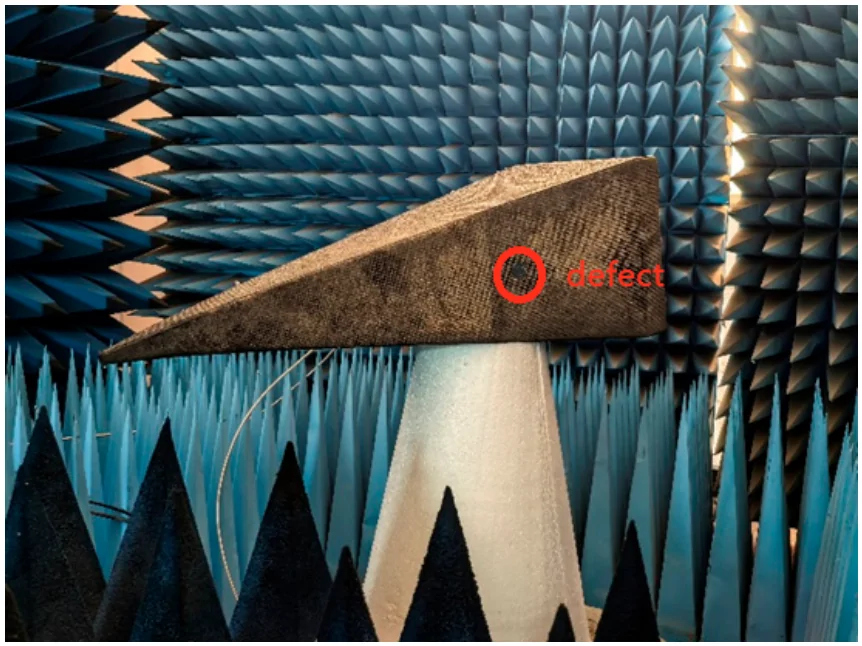
Coated conical specimen in the anechoic chamber. The red circle marks the localized coating defect.

A radar-absorbing-coated conical specimen with localized coating loss of 1 cm × 1 cm was used. Far-field measurements were performed in a microwave anechoic chamber, as shown in Figure 12. The intact and damaged states used the same frequencies, antenna and polarization configuration, target mounting reference and angular-scan settings, with a common calibration reference for comparing complex scattering responses.

Three reference scenes are measured with the actual antenna and connecting chain: free space, a metal plate, and a same-material plate provide background, strong-reflection, and material references, respectively. Both states use the same reference scenes, frequency, polarization, and matched geometry. Background subtraction and metal-reference normalization are expressed as:

(44) $$S_{q}^{\mathrm{cal}}\left(f\right)=\frac{S_{q}(f)-S_{\mathrm{air}}(f)}{S_{\mathrm{metal}}(f)-S_{\mathrm{air}}(f)},q\in \left\{0,d,\mathrm{mat}\right\}.$$

Here, *S_q_*(*f*) is the measured complex response of state q, and *S_air_*(*f*) and *S_metal_*(*f*) are the measured free-space and metal-plate responses, respectively. The quantity $S_{q}^{\mathrm{cal}}$(*f*) is the corresponding background-subtracted, metal-reference-normalized response. The index *q* ∊ {0, *d*, *mat*} denotes the intact state, damaged state, or same-material plate.

**Figure 13:**
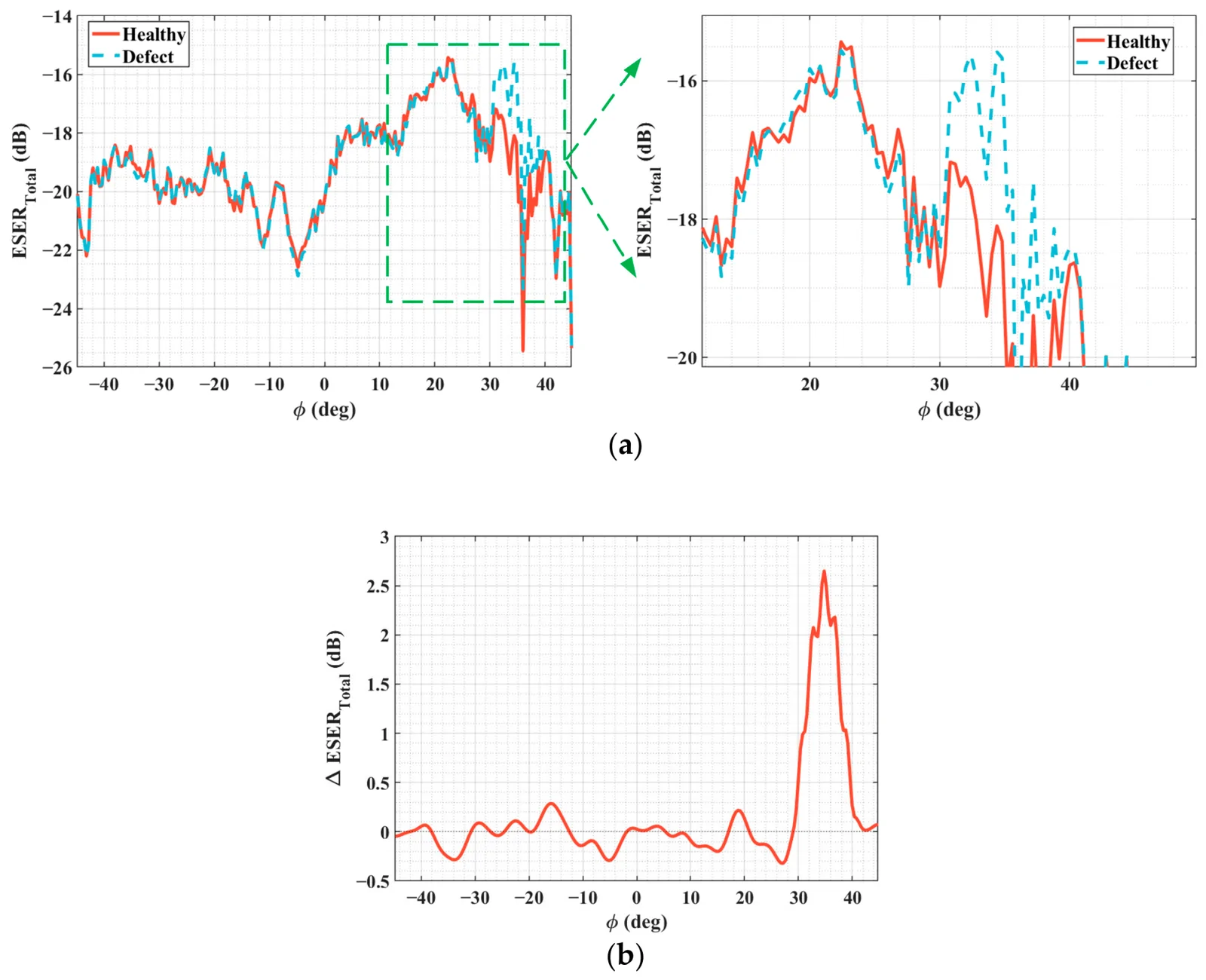
Measured far-field angular-domain responses at 10 GHz: (**a**) logarithmic ESER for the intact and damaged states, with an enlarged view of the region outlined in green; (**b**) azimuth difference factor relative to the intact reference.

The far-field acquisition covered 8–12 GHz at 0.01 GHz intervals, giving 401 frequency points, and azimuths from −44.8° to 44.8° at 0.4° intervals, giving 225 angular positions. Figure 13 uses the squared magnitude of the complex response at 10 GHz to calculate logarithmic ESER for both states and the azimuth difference factor.

The experimental far-field test results are shown in Figure 13.

The intact and damaged states exhibit similar logarithmic ESER values over most of the measured angular range. A pronounced difference occurs between approximately 30° and 40°, with a maximum increase of about 2.6 dB near 35°. This localized angular response is consistent with a damage-induced change in the coherent scattering of the coated specimen.

**Figure 14:**
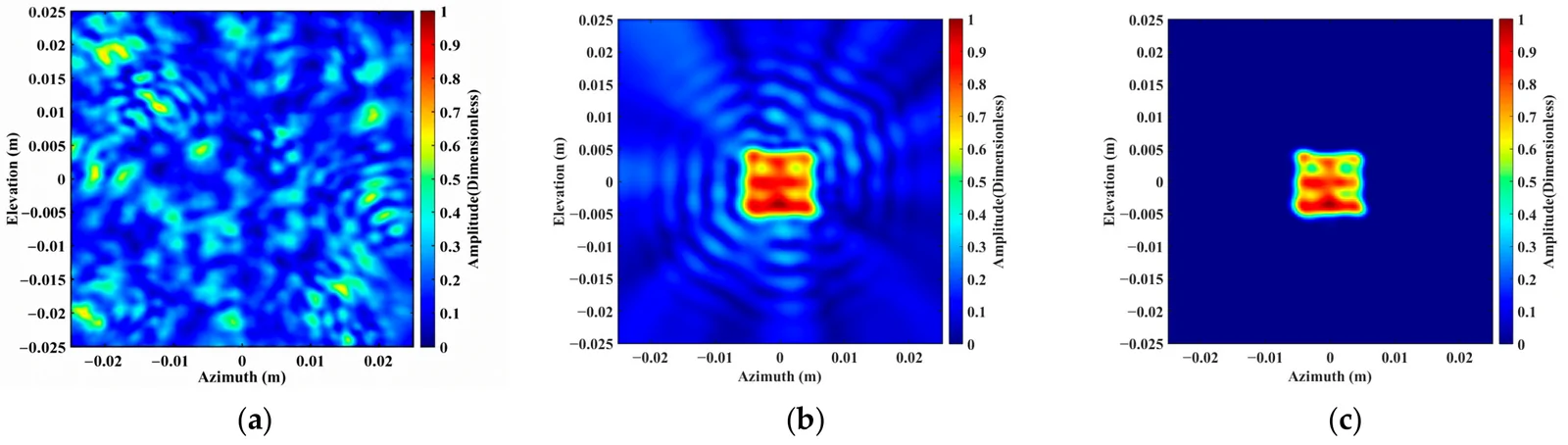
Local near-field imaging of the coated conical specimen at 40 GHz: (**a**) image of the intact state; (**b**) image of the state with localized coating loss; (**c**) normalized differential-response map used for equivalent-dimension extraction.

A two-dimensional near-field scan was performed over the selected 5 cm × 5 cm area. The acquisition band was 26.5–40 GHz with a 0.01 GHz frequency step. Each scan direction contained 51 positions at 1 mm intervals, giving 2601 spatial positions. Figure 14 presents single-frequency imaging by coherently summing the complex responses at 40 GHz using Equation (30). Both states used the same scan coordinates, amplitude scale, phase reference and imaging parameters, and their complex difference was calculated using Equation (32).

For the localized 10 mm × 10 mm coating loss on the conical specimen, the equivalent electromagnetic length and width are 10.92 mm and 10.63 mm, with deviations of 9.2% and 6.3%, respectively, from the nominal side lengths. This result demonstrates the integration of two-stage measurement and equivalent-scale extraction for the specimen, frequency and reference-matching conditions examined; the deviations are specific to this experimental case.

The parameter extraction results are shown in Table 3.

## 4. Discussion

### 4.1. Analysis of the Algorithm’s Defect Detection Capability

**Figure 15:**
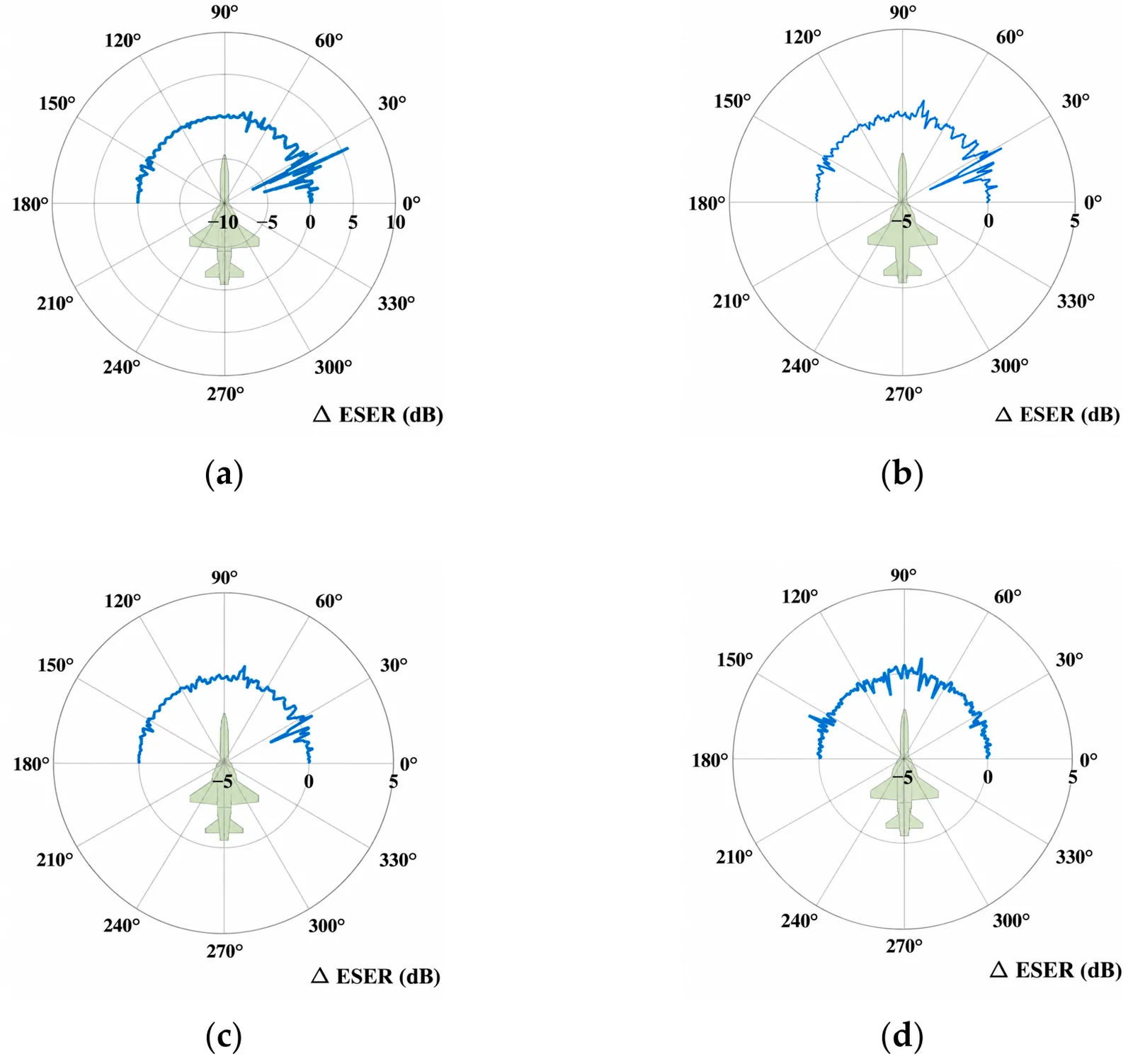
ΔESER for different defect sizes: (**a**) defect size 0.2λ; (**b**) defect size 0.16λ; (**c**) defect size 0.12λ; (**d**) defect size 0.08λ.

Using the model, materials and observation geometry in Section 3.1, impact defects with four discrete feature scales of 0.2*λ*, 0.16*λ*, 0.12*λ* and 0.08*λ* are compared, where *λ* is the free-space wavelength at the screening frequency. Equations (14)–(16) define the angular-anomaly criterion: the absolute azimuth difference factor exceeds the robust threshold over at least two consecutive angular samples. Figure 15 compares the anomaly responses at these discrete scales.

In Figure 15, the anomaly responses are clearer at 0.2*λ* and 0.16*λ*; defect-related changes remain discernible at 0.12*λ*, whereas the indication weakens substantially at 0.08*λ*. Approximately 0.12*λ* is therefore treated only as a condition-dependent reference scale among the discrete cases examined, with discernibility referring to the angular responses shown. It does not denote near-field spatial resolution, a universal detection limit or a performance level at a specified probability of detection.

### 4.2. Detection Efficiency Analysis

At the same frequency of 32 GHz and scan spacing of 2 mm, including both endpoints, a 0.9 m × 0.6 m plane contains 451 × 301 = 135,751 positions, giving 543,004 positions for four planes. *A* 0.2 m × 0.2 m local plane contains 101 × 101 = 10,201 positions, giving 20,402 positions for two planes and a 96.24% reduction in near-field positions. The 0.2 m window is used for this efficiency estimate and is distinct from the 0.16 m simulation aperture in Table 1. With the same average motion and acquisition time per position, this percentage also describes the reduction in the near-field point-by-point acquisition time.

To distinguish near-field sampling savings from total inspection time, the two workflows are expressed as:

*T_full_* = *T_common_* + *T_NF_*_,*full*_ + *T_proc_*_,*full*_, (45)

*T_two_* = *T_common_* + *T_far_* + *T_switch_* + *T_NF_*_,*local*_ + *T_proc_*_,*two*_. (46)

Here, *T_common_* denotes reference acquisition, calibration and common preparation; *T_far_* is the far-field angular acquisition time; *T_switch_* is the time for target or probe adjustment between stages; and *T_NF_* and *T_proc_* denote near-field acquisition and processing times, respectively. The 96.24% figure applies only to the near-field sampling configuration above; total time savings also depend on far-field acquisition, transition and processing overheads.

### 4.3. Sensitivity to Noise and Observation Geometry

**Figure 16:**
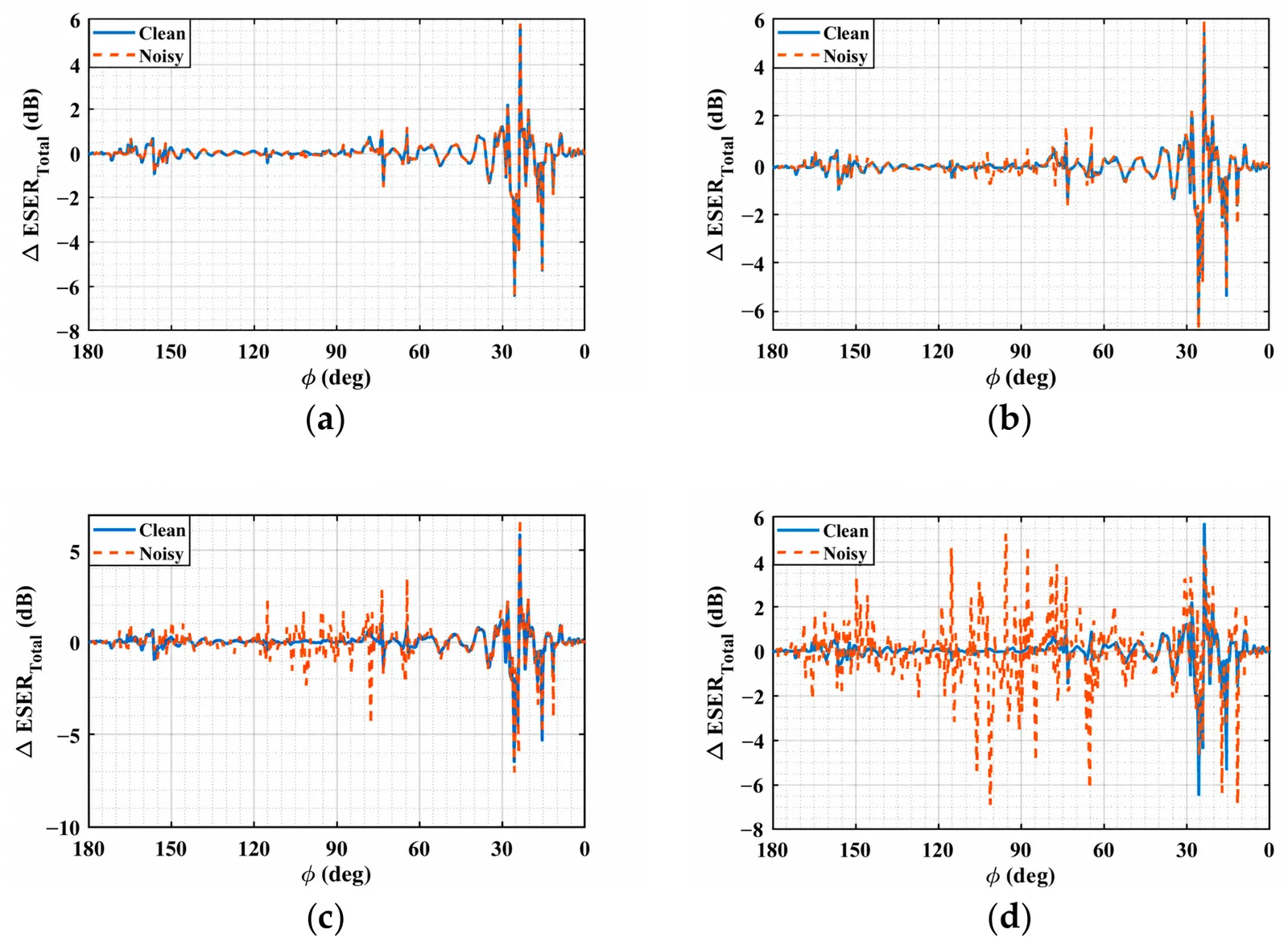
Comparison of the azimuth difference factor with and without added noise: (**a**) SNR = 60 dB; (**b**) SNR = 50 dB; (**c**) SNR = 40 dB; (**d**) SNR = 30 dB.

Far-field scattering data were perturbed with random noise at SNRs of 30, 40, 50, and 60 dB. Figure 16 compares the resulting azimuth-difference-factor curves with the corresponding noise-free curve.

As the SNR decreases from 60 dB to 30 dB, random fluctuations in the azimuth-difference curve increase and the anomaly peaks become less distinct. Figure 16 characterizes the sensitivity of angular-domain screening to random noise; peak and interval-boundary interpretation should account for the corresponding noise level.

**Figure 17:**
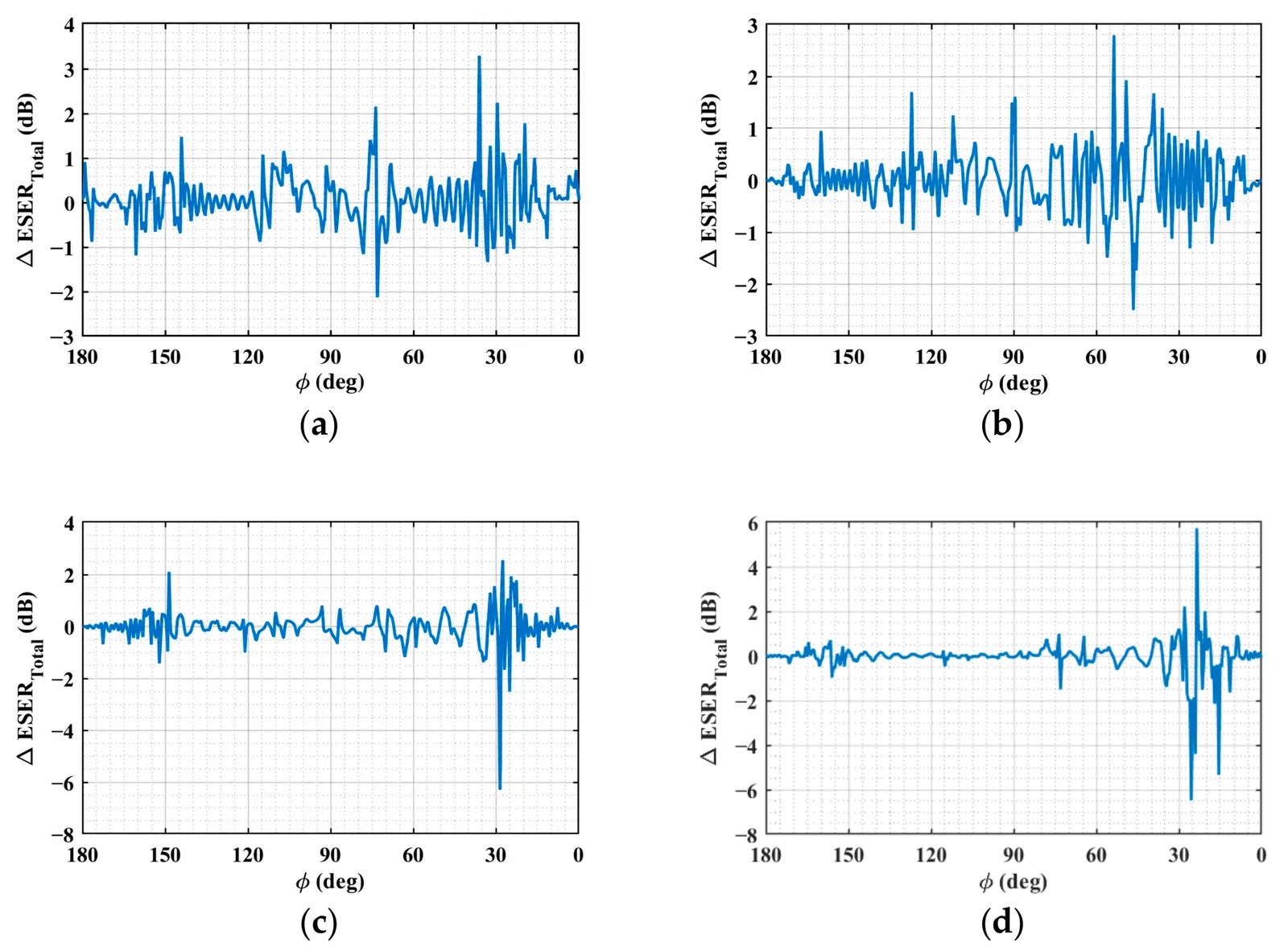
ΔESER at different incident angles: (**a**) θ = 60°; (**b**) θ = 70°; (**c**) θ = 80°; (**d**) θ = 90°.

Regarding the incident angle, this paper further changes the incident elevation angle, setting θ = 60°, 70°, 80°, and 90, respectively, to examine the sensitivity of ΔESER to changes in incident geometry. The relevant comparison results are shown in Figure 17.

At incident elevation angles of 60°, 70°, 80° and 90°, the selected cases retain discernible defect-related angular differences. Figure 16 and Figure 17 assess sensitivity to random noise and observation geometry, respectively, rather than providing a comprehensive robustness assessment. Anechoic far-field measurements, common calibration and amplitude/phase references, and matched mounting and scan coordinates were used to reduce systematic measurement errors in the intact/damaged comparison (Section 3.2). Residual effects of antenna positioning, phase drift, calibration, environmental reflections and intact/damaged registration remain and limitations of the present demonstration.

### 4.4. Engineering Applicability Analysis

The simplified aircraft-like model tests the method against a complex geometric background, while the coated conical specimen tests the measurement workflow on a controlled curved surface. They provide numerical demonstration and curved-surface measurement evidence, respectively. Application to actual aircraft skins requires inspection parameters to be adapted to the structural geometry, coating material, curvature, probe accessibility and reference-state configuration.

From an engineering implementation perspective, far-field angular-domain scattering data can be acquired through turntable testing or multi-station antenna scanning, and the priority observation direction for local scanning can be selected based on the anomaly location. Subsequently, a vector network analyzer, broadband antenna, and two-dimensional scanning platform can be used to perform local near-field imaging of the candidate region. This process can narrow the spatial range of near-field point-by-point scanning, reduce the number of near-field sampling points, and provide conditions for further engineering field verification.

Table 4 summarizes representative microwave-based approaches for detecting defects in radar-absorbing materials and coatings, comparing their reported measurement configurations and spatial outputs. Performance metrics should be interpreted in relation to the respective defect types and datasets.

The present method directly compares intact and damaged complex scattering responses, with experimental bands of 8–12 GHz for far-field measurements and 26.5–40 GHz for near-field acquisition. Each state includes 225 angular positions and 2601 near-field positions over a 5 cm × 5 cm area; the displayed near-field image uses 40 GHz. Impact, coating loss and bulging are examined numerically, while localized coating loss is tested experimentally. Outputs are a local center and equivalent electromagnetic dimensions. *A* matched intact reference and known structural partition are inputs to the workflow. Its contribution is the use of far-field screening to plan near-field coverage before local characterization; the sampling comparison at a common spacing is given in Section 4.2. Mechanical sampling positions and thermal-image pixels represent different quantities and are not compared directly.

## 5. Conclusions

This paper addresses the limitations of anomaly location capabilities and low efficiency of full-domain near-field scanning in large-scale inspection of radar-absorbing coatings on aircraft skin. A multiscale electromagnetic detection method combining far-field angular-domain screening and local near-field imaging is proposed and validated using an aircraft model and a measured model. The main work and conclusions are as follows:

(1) An angular domain scattering response analysis method for radar-absorbing coating damage was established. An azimuth difference factor was constructed by comparing the ESER of defect-free and damaged states, and continuous anomaly angular domains and the main anomaly azimuth were extracted. Combined with the coherent superposition relationship of complex scattering fields, it was shown that damage may cause both local scattering enhancement and destructive effects, thus providing a theoretical basis for using difference responses rather than single scattering amplitudes for anomaly screening.
(2) An anomaly angular domain-guided local near-field candidate region planning method was established. The main anomaly azimuth was used to determine the priority observation direction for local scanning, and the position and orientation of the candidate scanning plane were determined by combining target geometry, measurement distance, and prior knowledge of the detection area; the anomaly angular domain was used to constrain the azimuth coverage of the candidate region. Within the candidate window, a back-projection method is used to complete local two-dimensional imaging. The centroid of the differential response and the equivalent electromagnetic length and width are extracted using multithreshold analysis and squared-magnitude-weighted central second moments, linking far-field candidate screening to local spatial localization and equivalent-scale characterization.
(3) Numerical analysis demonstrates angular anomalies and local near-field responses for impact, coating loss and bulging. For rectangular coating loss, the equivalent electromagnetic length and width deviate from the nominal side lengths by 8.6% and 2.3%. The experiment demonstrates two-stage measurement and equivalent-scale extraction for localized 1 cm × 1 cm coating loss on the conical specimen, with corresponding deviations of 9.2% and 6.3%. Approximately 0.12*λ* is a condition-dependent reference scale among the discrete numerical cases examined. The noise and incidence-angle analyses characterize angular-response sensitivity under the specified perturbations.

The method uses far-field screening to organize local near-field characterization and applies to inspection tasks with a matched intact reference, structural partition and common coordinate system. The results pertain to the investigated defect types and sizes, local curvatures and measurement configurations. Application to other coatings or actual aircraft skins requires scan and characterization parameters to be selected for their materials, geometry and sampling conditions.

## Figures and Tables

**Table 1:** Aircraft model near-field scan parameters.

Frequency (GHz)	Scan Area (m × m)	Scan Interval (mm)	Lifting Height (m)
32	0.16 × 0.16	2.286	0.15

**Table 2:** Equivalent electromagnetic dimensions of different defects and comparison with nominal geometric scales.

Defect Type	Nominal Geometric Reference	Equivalent Electromagnetic Length/cm	Equivalent Electromagnetic Width/cm	Length Deviation/%	Width Deviation/%
Impact	0.5 cm radius circle	1.093	0.926	9.3	7.4
Coating loss	1 cm × 1 cm rectangle	1.086	0.977	8.6	2.3
Bulging	0.5 cm radius circle	0.965	0.934	3.5	6.6

**Table 3:** Equivalent electromagnetic dimensions extracted for the conical specimen.

Defect Type	Nominal Dimensions	Equivalent Electromagnetic Length/cm	Equivalent Electromagnetic Width/cm	Length Deviation/%	Width Deviation/%
Coating loss	10 mm × 10 mm	1.092	1.063	9.2	6.3

**Table 4:** Representative microwave-based approaches for detecting defects in radar-absorbing materials and coatings.

Method	Measurement Modality	Reported Frequency (GHz)	Spatial Characterization	Paired Intact-State Measurement	Experimental Validation	Scanning/Acquisition Strategy
Wu et al. [14]	Microwave imaging and object detection	4–18 (spot-frequency operation)	Damage classes and bounding boxes	Not used as a detector input	RAM specimens	Two-dimensional multiprobe array
Tu et al. [16]	Microwave lock-in/long-pulse thermography	5.8	Thermal maps of debonding	-	RAM debonding specimens	Area heating and thermal imaging; 90 × 90 mm field
Fang et al. [18]	Microwave-image clutter suppression and detection	4–18 (spot-frequency operation)	Pixel-wise retention and damage bounding boxes	Not used as a model input	Measured curved-RAM images	Two-dimensional cross-array acquisition
He et al. [19]	Resonant-cavity microwave thermography	-	Pixel-level debonding indications	Not used; same-specimen thermal contrast	RAM-coated CFRP specimens	Cavity heating and full-field cooling images
This work	Far-field scattering and local near-field imaging	8–12/26.5–40 (far/near field)	Angular candidates, centroid and equivalent dimensions	Required	Coated cone with localized coating loss	Geometry-guided angular screening and local planar scanning

## Data Availability

The raw/processed data required to reproduce these findings cannot be shared at this time as the data also forms part of an ongoing study.
